# Should Schlemm Canal-Based MIGS Be Combined with Cataract Surgery in Patients Receiving Topical Glaucoma Therapy? A Cataract Surgeon-Oriented Review

**DOI:** 10.3390/jcm15145503

**Published:** 2026-07-14

**Authors:** Suguru Nakagawa, Toshikatsu Kaburaki, Kiyoshi Ishii

**Affiliations:** 1 Department of Ophthalmology, Jichi Medical University Saitama Medical Center, Saitama 3300834, Japan; 2 Department of Ophthalmology, Jichi Medical University, Tochigi 3290498, Japan; 3 Department of Ophthalmology, Saitama Red Cross Hospital, Saitama 3300081, Japan

**Keywords:** minimally invasive glaucoma surgery, cataract surgery, Schlemm canal, iStent, Hydrus microstent, Kahook Dual Blade, Tanito microhook, open-angle glaucoma, refractive outcomes, patient selection

## Abstract

**Background/Objectives**: Schlemm canal-based minimally invasive glaucoma surgery (MIGS) can be combined with cataract surgery, but topical glaucoma therapy alone is not a sufficient indication for adding MIGS. This review addresses when cataract surgery alone may be sufficient, when combined cataract surgery and MIGS may be appropriate, and when filtration surgery should be considered. **Methods**: This narrative review used a targeted PubMed/MEDLINE search of English-language literature published from January 2000 to May 2026 to support source identification and reference selection. The review focused on cataract surgery combined with Schlemm canal- or trabecular meshwork-targeted MIGS, including stent-based Schlemm canal procedures and trabeculotomy/goniotomy-based procedures, medication burden, ocular surface disease, refractive and visual outcomes, corneal endothelial safety, complications, angle visibility, guideline-based decision-making, and patient selection. Final references were selected according to clinical relevance to cataract-surgeon decision-making and evidence priority, with emphasis on guidelines, systematic reviews or meta-analyses, randomized or prospective comparative studies, pivotal or long-term studies, large real-world or post-market studies, and clinically informative safety, refractive, endothelial, imaging, or complication-related studies. **Results**: Stent-based Schlemm canal procedures and trabeculotomy/goniotomy-based procedures can provide additional IOP and medication reduction compared with cataract surgery alone in selected eyes with mild-to-moderate open-angle glaucoma. Stent-based procedures generally have a lower hyphema risk, whereas trabeculotomy/goniotomy-based procedures may provide comparable or greater IOP reduction in selected eyes but are associated with more frequent hyphema. In normal-tension glaucoma or low-baseline-IOP eyes, the expected benefit is often medication reduction or modest IOP lowering rather than reliable achievement of very low target IOP. Available refractive evidence remains limited and procedure-specific, but suggests that major refractive instability is uncommon in appropriately selected eyes. **Conclusions**: The decision to combine Schlemm canal-based MIGS with cataract surgery should be goal-directed rather than based solely on the presence of topical therapy. Practical selection should integrate glaucoma subtype, disease stage, baseline and target IOP, expected phacoemulsification-only IOP reduction, medication burden, ocular surface status, adherence, angle visibility, endothelial reserve, refractive objectives, and future filtration surgery options.

## 1. Introduction

Cataract and glaucoma commonly coexist in elderly patients, and cataract surgeons frequently encounter patients who are already receiving topical glaucoma medications at the time of cataract surgery. In such cases, the practical preoperative question is not simply whether glaucoma is present, but whether cataract surgery alone is sufficient or whether Schlemm canal-based minimally invasive glaucoma surgery (MIGS) should be combined. The Japanese Glaucoma Society Guidelines emphasize that glaucoma treatment should be guided by disease type, disease stage, and an individualized target intraocular pressure (IOP) [1]. Cataract surgery itself can reduce IOP, particularly in eyes with narrow angles or angle-closure mechanisms in which lens extraction may deepen the anterior chamber and widen the angle [2,3,4]. In medically controlled open-angle glaucoma, however, the additional IOP-lowering effect of phacoemulsification alone may be modest and may not meaningfully reduce medication burden [5,6].

Medication burden is clinically relevant for cataract surgeons. Long-term topical glaucoma therapy can contribute to ocular surface disease, epithelial toxicity, reduced tolerability, poor adherence, and incorrect instillation technique [7,8,9,10,11,12,13,14]. Therefore, the potential benefit of adding MIGS at the time of cataract surgery should be interpreted not only as additional IOP reduction, but also as medication reduction, ocular surface improvement, and simplification of long-term glaucoma management.

Non-incisional alternatives should also be considered before adding an intraocular procedure. Selective laser trabeculoplasty (SLT) has long-term evidence supporting IOP and medication reduction with a favorable safety profile in primary open-angle glaucoma [15]. Therefore, when the main goal is modest IOP lowering or reduction in medication burden, SLT may be an appropriate option before or instead of combined cataract surgery and MIGS in selected eyes.

Among the broad spectrum of MIGS procedures, this review focuses on Schlemm canal-based MIGS combined with cataract surgery, including stent-based Schlemm canal procedures such as iStent and Hydrus microstent, and trabeculotomy or goniotomy procedures such as Kahook Dual Blade (KDB) goniotomy and Tanito microhook ab interno trabeculotomy. These procedures are particularly relevant to cataract surgeons because they can be performed through a clear corneal incision or a small additional incision at the time of phacoemulsification and generally preserve the conjunctiva for possible future filtration surgery [16,17,18,19,20,21].

From a cataract surgeon’s perspective, the central issue differs from that emphasized in many device-centered or glaucoma-specialist-oriented reviews. The question is not only how much MIGS lowers IOP, but also whether it can reduce medication burden without compromising the fundamental goals of cataract surgery: refractive predictability, minimal surgically induced astigmatism, rapid visual rehabilitation, and preservation of visual quality. This perspective is especially important when toric intraocular lenses or strict refractive targets are considered, when ocular surface disease affects biometry, or when early postoperative hyphema or corneal edema may delay visual recovery.

Accordingly, this narrative review addresses a practical clinical question: should Schlemm canal-based MIGS be added when performing cataract surgery in patients using IOP-lowering medications? By focusing on glaucoma subtype, disease stage, individualized target IOP, medication burden, ocular surface status, angle visibility, refractive stability, visual outcomes, and the potential need for future filtration surgery, we propose a cataract surgeon-oriented decision framework for selecting cataract surgery alone, cataract surgery combined with MIGS, or filtration surgery. The proposed cataract surgeon-oriented decision framework is discussed in the practical algorithm section.

## 2. Literature Search and Reference Selection

This article was designed as a narrative review rather than a systematic review or meta-analysis. PubMed/MEDLINE was searched for English-language articles published between January 2000 and May 2026 that were relevant to cataract surgery combined with Schlemm canal-based or trabecular meshwork-based minimally invasive glaucoma surgery. PubMed/MEDLINE was the only bibliographic database searched; Scopus and Web of Science were not used.

The literature search used structured PubMed/MEDLINE domains for the main clinical evidence base and contextual or known-item checks for broader supporting domains. Structured domains addressed cataract-combined Schlemm canal-based or trabecular meshwork-based MIGS, review-level evidence, high-level evidence on cataract surgery or lens extraction and IOP reduction, ocular surface disease and topical glaucoma therapy, medication adherence and treatment burden, safety, complications, corneal endothelial outcomes, and refractive, visual-function, axial-length, astigmatic, toric-IOL, and patient-reported outcomes. Guideline and landmark evidence, selective laser trabeculoplasty, anterior segment imaging, uveitic glaucoma, device labeling, regulatory sources, and reviewer-suggested or known-item references were handled as contextual checks rather than as formal screening denominators. The exact PubMed/MEDLINE search strings, record counts, and whether each domain was used as a structured search domain or a contextual check are provided in Supplementary Table S2.

References were selected according to direct clinical relevance to the review question and evidence priority. Priority was given to glaucoma guidelines, systematic reviews and meta-analyses, American Academy of Ophthalmology reports, randomized or prospective comparative studies, pivotal or long-term studies, large real-world or post-market studies, and clinically informative studies addressing safety, complications, refractive outcomes, endothelial effects, imaging findings, medication burden, ocular surface disease, adherence, or patient selection. Relevant studies cited in the references of retrieved articles were also considered. Device-labeling and regulatory sources were added when necessary to provide clinically relevant device-specific information.

**Figure 1:**
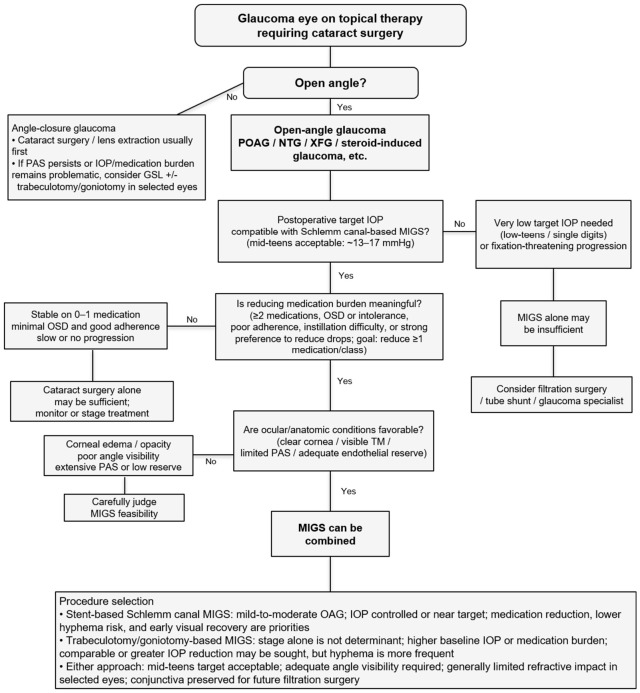
Practical decision algorithm for selecting cataract surgery alone, stent-based Schlemm canal MIGS, trabeculotomy/goniotomy-based MIGS, or filtration surgery. The algorithm is intended as a pragmatic clinical framework rather than a rigid rule. The key early decision is not disease stage alone, but whether the individualized target IOP is compatible with Schlemm canal-based surgery. A mid-teens target IOP is operationalized as approximately 13–17 mmHg, whereas very low target IOP refers to low-teens or single-digit pressure, especially in eyes with fixation-threatening progression or high risk of central visual loss. Medication reduction is considered clinically meaningful when medication burden is high, such as use of two or more medications or medication classes, or when drop-related problems are clinically important even with fewer medications, including ocular surface disease or preservative-related toxicity, medication intolerance or allergy, poor adherence, instillation difficulty, or a strong preference to reduce drops, with a practical goal of reducing at least one medication or medication class. Both stent-based and trabeculotomy/goniotomy-based MIGS require adequate angle visibility and favorable ocular/anatomic conditions. In angle-closure glaucoma, cataract surgery or lens extraction is generally considered first, and GSL with or without trabeculotomy/goniotomy may be considered in selected eyes when PAS, IOP elevation, or medication burden remains problematic. GSL = goniosynechialysis; IOP = intraocular pressure; MIGS = minimally invasive glaucoma surgery; NTG = normal-tension glaucoma; OAG = open-angle glaucoma; OSD = ocular surface disease; PAS = peripheral anterior synechiae; POAG = primary open-angle glaucoma; TM = trabecular meshwork; XFG = exfoliation glaucoma.

Because this was a narrative review, formal PRISMA-style record identification, screening, exclusion counting, risk-of-bias assessment, and meta-analysis were not performed. Retrieved PubMed/MEDLINE records were therefore not used as systematic screening denominators. To provide exact source-category information appropriate for this narrative review, the final reference list comprised 100 sources, including 97 PubMed-indexed peer-reviewed or guideline-based sources and 3 device-labeling or regulatory sources. Supplementary Figure S1 summarizes the literature-identification and reference-selection framework. Figure 1 presents the practical cataract surgeon-oriented decision algorithm, and Supplementary Table S2 summarizes the structured PubMed/MEDLINE search domains and contextual checks used to support the review. Supplementary Table S2 also maps the final cited reference numbers to the structured search domain or contextual check through which each source was identified or prioritized; because the domains were intentionally overlapping, this mapping is descriptive rather than a PRISMA-style screening denominator.

## 3. Overview of Schlemm Canal-Based MIGS Combined with Cataract Surgery

Minimally invasive glaucoma surgery is not a single procedure but rather a broad concept that includes several surgical approaches with different anatomical targets, mechanisms of action, and risk profiles [16,18,19,20]. In general, MIGS procedures can be classified according to their principal outflow pathway: trabecular or Schlemm canal-based procedures, subconjunctival filtration procedures, suprachoroidal outflow procedures, and cyclodestructive or aqueous production-modulating procedures. Among these, Schlemm canal-based MIGS is particularly relevant to cataract surgeons because many of these procedures can be performed ab interno through a clear corneal incision and can be combined relatively easily with phacoemulsification.

The present review focuses on Schlemm canal-based MIGS combined with cataract surgery. For practical purposes, these procedures can be divided into two major categories: stent-based Schlemm canal procedures and trabeculotomy or goniotomy-based procedures (Table 1). Stent-based Schlemm canal procedures include trabecular micro-bypass stents such as iStent and iStent inject, and a Schlemm canal microstent such as the Hydrus microstent. These devices are designed to bypass the trabecular meshwork and facilitate aqueous humor drainage into Schlemm canal and the distal collector channel system. In contrast, trabeculotomy- or goniotomy-based procedures, including KDB goniotomy and Tanito microhook ab interno trabeculotomy, reduce trabecular outflow resistance by incising or excising the trabecular meshwork.

This distinction is clinically important because the balance between efficacy and safety differs among procedures. Trabecular micro-bypass stent procedures are generally considered less traumatic and are associated with relatively low rates of postoperative inflammation and hyphema [22,23,24,25,35,36,60]. They are particularly suitable when medication reduction and maintenance of a favorable safety profile are the primary goals. By contrast, trabeculotomy/goniotomy-based procedures may provide IOP reduction comparable to or greater than that of trabecular micro-bypass stents in selected eyes, but they are more frequently associated with postoperative hyphema because the trabecular meshwork and Schlemm canal are directly opened [25,36,41,42,43,44,45,46,47,48,49,50,51].

All Schlemm canal-based procedures share an important physiological limitation. Because these surgeries enhance conventional aqueous outflow, postoperative IOP is influenced by distal outflow resistance and episcleral venous pressure, which is commonly approximately 8–10 mmHg [61]. Therefore, Schlemm canal-based MIGS is physiologically unlikely to achieve sustained single-digit IOP in most eyes. This point is particularly important in NTG or low-baseline-IOP eyes: the expected benefit should usually be framed as medication reduction or modest IOP reduction rather than reliable achievement of very low target IOP. These procedures can be appropriate when IOP in the mid-teens (approximately 13–17 mmHg) is acceptable, but they are usually insufficient for advanced or rapidly progressive glaucoma requiring low-teens or single-digit IOP.

Another key surgical requirement is adequate intraoperative angle visualization. Unlike cataract surgery alone, Schlemm canal-based MIGS requires visualization of the nasal angle structures using gonioscopy or a surgical gonioprism. Corneal edema, corneal opacity, endothelial disease, shallow anterior chamber, poor pupil dilation, hyphema, or peripheral anterior synechiae may reduce angle visibility and make the procedure technically difficult or unsafe [62]. Thus, the feasibility of MIGS should be assessed not only from the standpoint of glaucoma severity and target IOP, but also from the standpoint of anterior segment anatomy and intraoperative visibility.

Accordingly, procedure selection should consider not only the expected magnitude of IOP reduction, but also medication burden, angle visualization, refractive goals, early visual recovery, and preservation of the conjunctiva for possible future filtration surgery. The overall decision framework for selecting cataract surgery alone, combined Schlemm canal-based MIGS, or filtration surgery is summarized in Figure 1.

## 4. IOP- and Medication-Lowering Outcomes After Combined Cataract Surgery and MIGS

The IOP-lowering effect of cataract surgery alone is well recognized, but the magnitude of this effect is variable. In eyes with ocular hypertension or open-angle glaucoma, phacoemulsification alone has been reported to reduce IOP by approximately 3–4 mmHg in some cohorts, whereas larger reductions, often approximately 8–9 mmHg, have been reported in eyes with angle-closure mechanisms or higher baseline IOP [2,3,4,63,64,65]. In medically treated open-angle glaucoma, however, cataract surgery alone may not achieve sufficient IOP reduction or medication discontinuation [5,6,64]. Combined MIGS has therefore been developed as a strategy to enhance IOP reduction and reduce medication burden while maintaining a relatively favorable safety profile; this overall adjunctive benefit is also supported by recent systematic reviews and meta-analyses, randomized comparative evidence, and an American Academy of Ophthalmology report on trabecular procedures combined with cataract surgery [17,21,34,66]. Selected representative studies relevant to IOP- and medication-lowering outcomes are provided in Table S1.

### 4.1. Stent-Based Schlemm Canal Procedures

Trabecular micro-bypass stents have been evaluated in several prospective studies and randomized clinical trials. The first-generation iStent was one of the earliest devices studied in combination with cataract surgery. In a randomized trial, Samuelson and colleagues demonstrated that implantation of a trabecular micro-bypass stent at the time of phacoemulsification resulted in a higher proportion of eyes achieving target IOP reduction without medications compared with cataract surgery alone [22]. This study established the concept that adding a trabecular micro-bypass procedure to cataract surgery can provide incremental glaucoma control beyond lens extraction alone.

Subsequent studies evaluated second-generation trabecular micro-bypass stents. The pivotal trial of iStent inject combined with cataract surgery showed significant IOP reduction and medication reduction compared with cataract surgery alone [23]. Because iStent inject allows implantation of multiple stents, it was designed to increase the probability of accessing functioning collector channels and thereby improve aqueous outflow. In clinical practice, iStent and iStent inject are often selected for eyes with mild-to-moderate open-angle glaucoma in which medication reduction and a favorable safety profile are major goals.

The Hydrus microstent differs from iStent in that it not only bypasses the trabecular meshwork but also scaffolds a segment of Schlemm canal. In the HORIZON trial, Hydrus microstent implantation combined with cataract surgery resulted in greater IOP reduction, greater medication reduction, and a lower rate of subsequent glaucoma surgery compared with cataract surgery alone [26]. Five-year follow-up data confirmed the durability of these benefits [27]. A secondary analysis of the HORIZON cohort suggested that Hydrus implantation may be associated with slower visual field progression than cataract surgery alone [28]. A systematic review and meta-analysis of randomized trials has also emphasized the importance of visual field outcomes when cataract surgery is performed with or without MIGS [67]. This observation may reflect greater IOP reduction, reduced medication burden, or improved IOP stability; however, the available evidence does not imply that Schlemm canal-based MIGS can substitute for filtration surgery in eyes with advanced glaucoma or very low target IOP. These findings support the clinical relevance of the Hydrus microstent not only as an IOP-lowering intervention, but also as part of a long-term glaucoma management strategy in appropriately selected eyes.

Comparative studies of standalone MIGS procedures have also provided useful context for understanding differences between iStent and Hydrus microstent. The COMPARE study evaluated Hydrus and iStent as standalone procedures and provided information regarding the relative efficacy and safety of stent-based Schlemm canal implants [29]. Although this study was not conducted in combination with cataract surgery, it helps contextualize device-specific differences within stent-based Schlemm canal approaches. Other long-term and comparative studies, including studies of stent-based Schlemm canal procedures combined with phacoemulsification, have further supported the safety and efficacy of iStent and Hydrus in clinical practice [30,31,32,68]. A recent randomized trial directly comparing cataract surgery alone with combined iStent inject W or KDB Glide further supports the need to distinguish trabecular micro-bypass stent and goniotomy-based procedures when considering cataract-combined MIGS [60]. These data help contextualize the results of pivotal randomized trials and show that stent-based Schlemm canal procedures have accumulated both trial-based and real-world evidence.

### 4.2. Trabeculotomy and Goniotomy-Based Procedures

Trabeculotomy- and goniotomy-based MIGS procedures reduce trabecular outflow resistance more directly by incising or removing the trabecular meshwork. KDB goniotomy uses a dual-blade design to excise a strip of trabecular meshwork, whereas Tanito microhook ab interno trabeculotomy incises the trabecular meshwork through an ab interno approach. These procedures do not require permanent device implantation and can be combined with cataract surgery through a corneal incision.

Clinical studies of KDB goniotomy combined with cataract surgery have reported meaningful reductions in IOP and/or medication burden [35,41,52,60]. However, in a randomized study of well-controlled mild-to-moderate glaucoma, adding KDB goniotomy showed no clear additional IOP- or medication-lowering benefit over phacoemulsification alone at 12 months [42]. Although many of these studies are retrospective and heterogeneous in design, they consistently suggest that trabeculotomy/goniotomy-based procedures are effective options for patients with mild-to-moderate glaucoma undergoing cataract surgery. Compared with trabecular micro-bypass stent procedures, trabeculotomy/goniotomy-based procedures may provide comparable or greater IOP reduction in selected eyes, but at the cost of more frequent postoperative hyphema.

Tanito microhook ab interno trabeculotomy has been widely reported from Japan and is particularly relevant when discussing Asian real-world data. In a large series, Tanito and colleagues reported favorable midterm outcomes after microhook ab interno trabeculotomy, including significant IOP and medication reduction [43]. Additional Japanese studies of KDB or Tanito microhook procedures combined with phacoemulsification have also reported sustained IOP and medication reduction [44,46]. Prospective data from other Asian populations have further supported the efficacy of Tanito microhook ab interno trabeculotomy combined with cataract surgery [45,47]. These data are important because they reflect outcomes in populations in which normal-tension glaucoma is common and baseline IOP may be lower than in many Western clinical trials.

Recent comparative and long-term data have further refined the interpretation of trabeculotomy/goniotomy-based MIGS. A systematic review and meta-analysis by Guedes and colleagues compared KDB goniotomy and iStent implantation, both combined with phacoemulsification, and suggested that KDB goniotomy may achieve higher surgical success and greater IOP reduction than first-generation iStent, whereas differences between KDB and iStent inject were less clear [36]. Long-term real-world studies have also reported sustained IOP and medication reduction after phaco-KDB over extended follow-up periods [48,49]. In addition, KDB goniotomy combined with cataract surgery has shown potential efficacy in normal-tension glaucoma, although the evidence remains limited and should not be overgeneralized [50]. These findings support the role of trabeculotomy/goniotomy-based MIGS as an effective option in selected eyes, while emphasizing the need to balance greater IOP reduction against the higher likelihood of early postoperative hyphema.

### 4.3. Japanese and Asian Real-World Evidence

Japanese data are particularly important for interpreting the role of MIGS in eyes with relatively low baseline IOP. Kanda and colleagues reported favorable clinical outcomes after phacoemulsification combined with first- and second-generation trabecular micro-bypass stents in Japanese patients [24]. Inatani and colleagues also reported two-year post-market surveillance outcomes of iStent inject W combined with phacoemulsification in Japanese open-angle glaucoma eyes, showing sustained reductions in IOP and medication burden [33]. A propensity score-matched Japanese study in eyes in which trabecular micro-bypass stent procedures could reasonably be considered reported similar postoperative IOP and medication-score reductions between trabecular micro-bypass stent procedures and microhook ab interno trabeculotomy, providing comparative data relevant to procedure selection [25].

However, the expected benefit of MIGS in Japanese patients may differ from that in Western trial populations. The Tajimi Study demonstrated that primary open-angle glaucoma is common in Japan and that a large proportion of affected eyes have IOP within the statistically normal range [69,70]. A recent systematic review of MIGS in normal-tension glaucoma similarly suggests that postoperative IOP reduction is often modest, whereas medication burden may decrease [71]. Therefore, in many Japanese patients, the absolute IOP reduction after MIGS may be smaller than that reported in studies enrolling patients with higher baseline IOP. In such eyes, medication reduction, improved ocular surface status, and simplification of long-term therapy may be more clinically meaningful than large IOP reduction alone.

Ocular surface and adherence considerations further support this view. Chronic topical therapy can contribute to ocular surface disease and medication intolerance [7,8,10,11,12,14,72]. Poor adherence is also common in glaucoma patients and may worsen with increasing treatment complexity [9,13,14,73,74]. Therefore, combined cataract surgery and MIGS may offer benefits beyond numerical IOP reduction by reducing medication burden, improving ocular surface status, supporting visual quality and satisfaction, and simplifying long-term management.

### 4.4. Interpreting IOP Reduction in the Context of Target IOP

When evaluating MIGS outcomes, postoperative IOP should be interpreted in relation to the individualized target IOP rather than as an isolated numerical reduction [1,55]. Practical thresholds are not absolute, but the following framework may help cataract surgeons. The key early decision is not disease stage alone, but whether the individualized target IOP is compatible with Schlemm canal-based surgery. If low-teens or single-digit IOP is required, particularly in the presence of fixation-threatening progression or high risk of central visual loss, Schlemm canal-based MIGS alone may be insufficient and filtration surgery, tube shunt surgery, staged surgery, or a glaucoma-specialist procedure should be considered. Conversely, if a mid-teens target IOP is acceptable and the angle can be safely visualized, Schlemm canal-based MIGS may be considered when medication reduction or additional IOP lowering is clinically meaningful.

Procedure selection should distinguish stent-based Schlemm canal MIGS from trabeculotomy/goniotomy-based MIGS. Stent-based Schlemm canal MIGS is best suited for eyes with mild-to-moderate open-angle glaucoma undergoing cataract surgery when IOP is medically controlled or near the individualized target, the desired postoperative IOP is compatible with mid-teens pressure, the angle can be safely visualized, and the main goals are medication reduction, ocular surface improvement, refractive stability, early visual recovery, and a lower hyphema risk. This interpretation is consistent with the device-specific indication and evidence base for stent-based Schlemm canal procedures, which primarily support use in mild-to-moderate open-angle glaucoma combined with cataract surgery.

By contrast, trabeculotomy/goniotomy-based MIGS should not be selected or excluded according to disease stage alone. These procedures may be considered across a broader range of disease stages when the individualized target IOP is compatible with mid-teens pressure, when the angle can be safely visualized, and when baseline IOP or medication burden provides room for clinically meaningful reduction. For example, an eye with more advanced visual field loss but IOP above 20 mmHg may still be a reasonable candidate for initial trabeculotomy/goniotomy-based surgery if the intended postoperative target is in the mid-teens and conjunctival preservation is desirable. However, if the target IOP is in the low teens or single digits, or if fixation-threatening progression requires very low IOP, filtration surgery, tube shunt surgery, or glaucoma-specialist surgery should be considered rather than relying on Schlemm canal-based MIGS alone.

Medication burden should also be operationalized rather than interpreted as the simple presence of any topical therapy. Combined cataract surgery and MIGS is more compelling when the patient uses two or more glaucoma medications or medication classes, or when drop-related problems are clinically important even with fewer medications, such as ocular surface disease or preservative-related toxicity, medication intolerance, poor adherence, difficulty with instillation, or a strong preference to reduce drops. A practical medication-reduction goal is reduction of at least one medication or medication class. Cataract surgery alone may be sufficient when the eye is stable on no or one medication, adherence is good, ocular surface disease is minimal, progression is absent or slow, and the expected IOP reduction from cataract surgery alone is likely to meet the individualized target IOP.

The expected magnitude of IOP reduction should be interpreted according to baseline IOP and surgical goal. In low-baseline-IOP or normal-tension glaucoma eyes, the main expected benefit of Schlemm canal-based MIGS is often medication reduction rather than large IOP lowering; additional IOP lowering, when achieved, is often modest, for example approximately ≤2–3 mmHg or <20% reduction [71]. In eyes with higher baseline IOP and suitable anatomy, Schlemm canal-based MIGS may provide clinically meaningful IOP and/or medication reduction in selected eyes [25,35,36,41,42,43,44,45,46,47,48,49,50,51,52,60], which may be operationalized in this framework as approximately ≥3 mmHg or ≥20% IOP reduction and/or reduction in one to two medications or medication classes. 

Anatomic suitability must be assessed before adding angle-based MIGS. Clear corneal media, adequate gonioscopic visualization of the nasal trabecular meshwork over the intended treatment area, an open angle or limited/manageable peripheral anterior synechiae (PAS), sufficient endothelial reserve, and stable zonules favor safe implantation or trabecular incision. Poor angle visibility, extensive PAS involving the intended treatment area, corneal edema or opacity, marked endothelial compromise, shallow anterior chamber, or unstable zonules should lower the threshold for abandoning MIGS or choosing an alternative strategy.

## 5. Refractive Outcomes After Schlemm Canal-Based MIGS Combined with Cataract Surgery

Refractive predictability is a central concern for cataract surgeons because modern cataract surgery is expected to provide both visual rehabilitation and accurate refractive outcomes. When glaucoma surgery is added, refractive outcomes may be affected by ocular surface disease, corneal biomechanics, axial length, anterior chamber depth, zonular status, postoperative IOP change, and procedure-specific wound healing.

The strength of evidence differs substantially by procedure. Direct refractive data are available for stent-based procedures, particularly iStent/iStent inject, including studies of surgically induced astigmatism, refractive prediction error, toric IOL use, and visual outcomes [37,38,39,40]. Data for trabeculotomy/goniotomy-based procedures, including KDB goniotomy, Tanito microhook ab interno trabeculotomy, and GATT are more heterogeneous and include both refractive-outcome and biometric studies [37,54,75]. Recent data on cataract surgery combined with GATT or KDB goniotomy suggest that refractive predictability is generally acceptable in selected eyes, but procedure-specific evidence remains limited and should be interpreted in relation to baseline IOP, magnitude of IOP reduction, and ocular biometric characteristics [54]. Evidence in eyes with angle closure, pseudoexfoliation, high preoperative IOP, or zonular weakness is more limited and should not be generalized from uncomplicated open-angle glaucoma cohorts [66,76,77,78].

Current evidence suggests that Schlemm canal-based MIGS combined with cataract surgery does not usually create a large refractive penalty in appropriately selected eyes. However, this conclusion should be interpreted cautiously because available studies are often retrospective, procedure-specific, and based on selected eyes with relatively favorable surgical conditions. The magnitude and timing of postoperative IOP reduction may also influence axial length and refractive outcome, especially after trabeculotomy-based procedures or in eyes with high preoperative IOP [75].

For toric IOL planning or strict refractive targeting, the procedure should be selected with refractive stability and the expected magnitude of IOP reduction in mind. Available evidence suggests that both stent-based procedures and trabeculotomy/goniotomy-based procedures can generally provide acceptable refractive predictability in selected eyes [37,38,39,40,54]. Trabeculotomy/goniotomy-based procedures may be considered when a greater IOP-lowering effect or medication reduction is sought, but surgeons should counsel patients regarding transient hyphema, early visual disturbance, and the possibility of small refractive shifts after substantial IOP reduction [25,36,41,42,43,44,45,46,47,48,49,50,58,75]. These considerations are summarized in Table 2.

## 6. Surgically Induced Astigmatism and Postoperative Visual Function

Surgically induced astigmatism is another key determinant of postoperative visual quality. Conventional filtering surgery can induce astigmatic changes through conjunctival peritomy, scleral flap creation, suturing, postoperative bleb formation, hypotony, and wound-healing changes near the superior cornea [57,58,59]. In contrast, Schlemm canal-based MIGS is usually performed through a clear corneal incision and is therefore expected to have a smaller direct corneal effect than trabeculectomy.

However, the evidence should be graded cautiously. Phaco-MIGS data suggest that trabecular micro-bypass stents and microhook ab interno trabeculotomy generally produce limited additional astigmatism in selected eyes [37]. Separately, comparative glaucoma-surgery data indicate that microhook ab interno trabeculotomy is less astigmatogenic than filtering surgery [53]. Yet most available data are retrospective or observational, and results may vary by incision location, gonioscopic manipulation, glaucoma subtype, and the magnitude of postoperative IOP reduction.

Postoperative visual function should also be interpreted broadly. Best-corrected visual acuity may improve after cataract surgery regardless of the MIGS procedure, but early vision can be affected by hyphema, corneal edema, IOP spikes, ocular surface disease, residual refractive error, and pre-existing glaucomatous damage [79]. Patient-reported outcomes are also important because the clinical value of combined cataract surgery and MIGS may include not only IOP reduction but also reduced medication burden, improved treatment convenience, and better glaucoma-specific quality of life [80,82]. In advanced glaucoma, even an anatomically successful cataract-MIGS procedure may not deliver the visual improvement expected by the patient.

Thus, available evidence suggests that the additional refractive impact attributable to Schlemm canal-based MIGS is limited in selected eyes. However, the evidence remains limited and procedure-specific. This issue is particularly relevant in selected eyes with relatively mild cataract and good preoperative unaided visual acuity, in which combined cataract surgery and MIGS is considered primarily for glaucoma-control or medication-reduction goals; in such eyes, small residual refractive errors may affect satisfaction when expectations for unaided vision are high.

## 7. Corneal Endothelial Safety

Corneal endothelial safety is another important consideration when MIGS is combined with cataract surgery. Although refractive stability and surgically induced astigmatism are important from the standpoint of cataract surgeons, endothelial cell loss (ECL) is also clinically relevant because corneal endothelial cells have limited regenerative capacity and excessive loss may lead to persistent corneal edema and delayed visual recovery. This issue is particularly important for procedures involving intracameral device implantation, as well as in eyes with shallow anterior chambers, pre-existing endothelial dysfunction, corneal guttata, or difficult intraoperative manipulation.

Available evidence suggests that stent-based Schlemm canal procedures are generally not associated with clinically prohibitive ECL when appropriately performed with cataract surgery. However, endothelial safety profiles may differ among devices and should be evaluated over the long term. Fea and colleagues reported that endothelial changes after combined cataract surgery and Hydrus implantation were comparable with those after phacoemulsification alone at 6 months [81]. Systematic and narrative reviews have emphasized that long-term endothelial surveillance remains important after MIGS, especially for implanted devices [84,85,86]. Ahmed and colleagues compared 5-year corneal endothelial safety profiles among several MIGS devices and reported that iStent inject had a favorable long-term endothelial safety profile, whereas device-specific differences were observed for Hydrus Microstent and CyPass Micro-Stent [87]. Although CyPass is not a Schlemm canal-based device, the CyPass experience, including 5-year endothelial cell loss data reported by Lass and colleagues and the subsequent voluntary market withdrawal of the device, underscores the broader importance of long-term endothelial assessment for implanted MIGS devices [88].

Therefore, although Schlemm canal-based MIGS is generally considered safe for the corneal endothelium, surgeons should not ignore endothelial risk. Preoperative specular microscopy may be useful in eyes with suspected endothelial compromise. In eyes with marked corneal edema, guttata, shallow anterior chamber, or poor intraoperative angle visibility, the indication for combined MIGS should be carefully individualized. Postoperative corneal edema should not be attributed automatically to cataract surgery alone; device position, intraoperative manipulation, and endothelial reserve should also be considered.

## 8. Axial Length Change, IOP Reduction, and Hyperopic Shift

A secondary refractive consideration in glaucoma surgery is the relationship between IOP reduction and axial length change. The eye is a biomechanical structure, and IOP contributes to ocular wall tension. When IOP decreases substantially, particularly after glaucoma surgery, axial length may shorten. Such axial length shortening may contribute to a hyperopic refractive shift, especially when IOL power has been calculated based on preoperative biometry obtained at a higher IOP.

This phenomenon has been recognized after trabeculectomy and glaucoma drainage device surgery. Francis and colleagues reported changes in axial length after trabeculectomy and glaucoma drainage device surgery, supporting the concept that large IOP reductions can alter ocular dimensions [58]. Refractive changes after glaucoma surgery, including trabeculectomy and other procedures, have also been reported in comparative studies [57]. These findings provide a mechanistic background for understanding why glaucoma surgery can influence postoperative refraction.

Although Schlemm canal-based MIGS is generally less likely than trabeculectomy to cause marked hypotony or large biometric shifts, axial length change may still become relevant when postoperative IOP reduction is substantial. This issue may be more relevant after trabeculotomy/goniotomy-based procedures, which can produce greater IOP reduction than stent-based Schlemm canal procedures in selected eyes.

Kanda and colleagues specifically investigated the relationship between IOP reduction after trabeculotomy, axial length, and IOL selection [75]. They reported that a large IOP decrease after trabeculotomy was associated with axial length shortening and could influence postoperative refraction. These findings suggest one possible mechanism by which combined cataract surgery and trabeculotomy-based procedures may produce mild refractive shifts even when corneal astigmatism and surgical wound effects are limited.

The clinical impact of this phenomenon depends on the magnitude of IOP reduction and the refractive target. In routine monofocal cataract surgery, a small hyperopic shift may not be clinically significant for many patients. When substantial IOP reduction is clinically required, glaucoma control usually takes priority over refractive precision; nevertheless, surgeons should recognize the possibility of axial length shortening and mild hyperopic shift during IOL planning and patient counseling.

Potential factors that may increase the relevance of axial length change include high preoperative IOP, the magnitude of postoperative IOP reduction, and baseline ocular biometric characteristics [58,75]. Glaucoma subtype may also be relevant. Pseudoexfoliation glaucoma can be associated with high IOP and zonular instability, while angle-closure eyes may have short axial length and shallow anterior chamber anatomy [76,77,78]. These features may interact with IOP-related biometric changes and affect refractive predictability.

At present, there is no universally accepted method for adjusting IOL power calculations based on anticipated IOP reduction after MIGS. Therefore, the most practical approach is risk recognition. In eyes with high preoperative IOP or planned trabeculotomy-based procedures expected to produce large IOP reduction, surgeons should recognize the possibility of postoperative hyperopic shift. Repeat biometry after IOP stabilization may be considered in selected cases if surgery is staged for other clinical reasons, although this is not applicable to most eyes undergoing simultaneous cataract surgery and MIGS.

**Figure 2:**
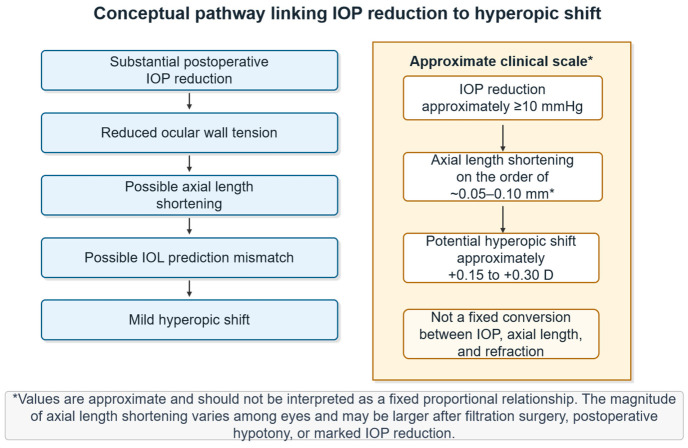
Conceptual pathway linking IOP reduction, axial length shortening, and mild hyperopic shift. The values shown are approximate and should not be interpreted as a fixed conversion between IOP, axial length, and refraction. IOL = intraocular lens; IOP = intraocular pressure.

This relationship can be conceptualized as substantial IOP reduction leading to reduced ocular wall tension, axial length shortening, and mild hyperopic shift (Figure 2). The values shown in Figure 2 are illustrative and should not be interpreted as fixed conversion factors. In the study by Kanda and colleagues, an IOP reduction of approximately 10 mmHg or greater was associated with axial length shortening of approximately 0.05–0.10 mm in some eyes [75]. Accordingly, axial length shortening should be regarded as a secondary refractive consideration when substantial IOP reduction is anticipated.

## 9. Complications and Surgical Considerations Relevant to Cataract Surgeons

Although Schlemm canal-based MIGS is generally considered safer and less invasive than conventional filtering surgery, it should not be regarded as free of complications (Table 3). The safety profile of MIGS differs according to procedure type, and cataract surgeons should understand procedure-specific risks before deciding whether to combine MIGS with phacoemulsification. In many eyes, complications are transient and manageable, but even temporary visual disturbance, hyphema, IOP spikes, or corneal edema may affect early postoperative recovery and patient satisfaction.

### 9.1. Hyphema and Blood Reflux

Hyphema is one of the most common findings after trabeculotomy/goniotomy-based MIGS. Because the trabecular meshwork is incised or excised, blood reflux from Schlemm canal and the collector channels is often observed intraoperatively or in the early postoperative period. In many cases, this reflux confirms communication with the conventional outflow pathway and resolves spontaneously without intervention. However, patients should be informed that early postoperative vision may be temporarily blurred.

The frequency and clinical relevance of hyphema differ between procedure categories. Trabecular micro-bypass stent procedures are generally associated with less hyphema than trabeculotomy/goniotomy-based procedures, whereas KDB goniotomy and Tanito microhook ab interno trabeculotomy more frequently produce visible postoperative blood in the anterior chamber [25,36,41,42,43,44,45,46,47,48,49,50]. Most cases are self-limited, but persistent or large hyphema can elevate IOP, delay visual recovery, and, in rare cases, raise concern for corneal blood staining. Anterior chamber washout may be required when hyphema is prolonged, visually significant, or associated with uncontrolled IOP.

Rarely, blood may migrate posteriorly and cause vitreous hemorrhage. Bothun and colleagues reported vitreous hemorrhage after trabeculotomy in aphakic eyes, illustrating that blood reflux may occasionally extend beyond the anterior chamber [96]. Although this complication is uncommon in routine adult combined cataract and MIGS surgery, cataract surgeons should be aware of the possibility, especially in eyes with abnormal anterior hyaloid integrity, aphakia, prior vitrectomy, zonular weakness, or complicated surgery.

### 9.2. Postoperative IOP Spikes

Transient IOP elevation can occur after both cataract surgery alone and combined Schlemm canal-based MIGS [16,18,19,20,24,25,33,35,36,41,42,43,44,45,46,47,48,49,50,52]. In the context of MIGS, IOP spikes may result from retained ophthalmic viscosurgical device, hyphema- or clot-related obstruction, postoperative inflammation, steroid response, or transient obstruction of the outflow pathway. Because hyphema and blood clot formation are more frequent after trabeculotomy- or goniotomy-based procedures, hyphema- or clot-related mechanisms are particularly relevant after these procedures [25,36,41,42,43,44,45,46,47,48,49,50]. Additionally, in a large phaco-LOT cohort, Goto and colleagues reported that long axial length was associated with a smaller early postoperative IOP decrease and a higher incidence of early IOP spikes, suggesting that eyes with long axial length may require closer early pressure monitoring after ab interno trabeculotomy [83].

Most IOP spikes can be managed with topical or systemic IOP-lowering medications, but they may be clinically important in eyes with advanced glaucoma or severely compromised optic nerves. This is one reason why Schlemm canal-based MIGS may be less appropriate as the sole glaucoma procedure for eyes requiring very low target IOP. In such patients, even short-term postoperative IOP elevation may be poorly tolerated.

### 9.3. Cyclodialysis, Hypotony, and Ciliochoroidal Detachment

Hypotony is less common after Schlemm canal-based MIGS than after trabeculectomy because the outflow pathway remains limited by the distal conventional outflow system and episcleral venous pressure [61]. However, hypotony can occur when cyclodialysis or abnormal communication between the anterior chamber and suprachoroidal space develops. This complication is particularly relevant to trabeculotomy/goniotomy-based procedures.

Clinically significant hypotony-related complications, including persistent hypotony associated with annular ciliochoroidal detachment, serous choroidal or retinal detachment, or cyclodialysis, have been reported after microhook ab interno trabeculotomy [92,93,94]. These reports indicate that although such events are uncommon, they can be clinically significant and may require gas tamponade, vitrectomy, or other surgical intervention.

The mechanism may involve procedure-related formation of a cyclodialysis cleft or abnormal communication between the anterior chamber and the suprachoroidal space during trabecular incision or angle manipulation [92,93,94]. Although specific risk factors are not well established, eyes with poor angle visibility, shallow anterior chamber, abnormal angle anatomy, peripheral anterior synechiae, or excessive intraoperative manipulation may be at higher risk. Therefore, the decision to perform trabeculotomy/goniotomy-based MIGS should include careful assessment of the angle anatomy and intraoperative visualization.

### 9.4. Corneal Transparency and Angle Visibility

Adequate angle visualization is essential for Schlemm canal-based MIGS. Unlike cataract surgery alone, these procedures require identification of the trabecular meshwork and accurate manipulation of the nasal angle. Corneal edema, corneal opacity, endothelial dysfunction, epithelial irregularity, shallow anterior chamber, poor ocular fixation, hyphema, or peripheral anterior synechiae can impair intraoperative gonioscopic visualization [62].

Poor angle visibility is not only a technical inconvenience but also a safety issue. If the trabecular meshwork cannot be clearly visualized, device malposition, incomplete trabeculotomy, iris trauma, Descemet membrane injury, cyclodialysis, or excessive bleeding may occur. Poor intraoperative visibility has been associated with technical difficulty during trabecular micro-bypass stent implantation and postoperative astigmatic change in a case report [62]. Although evidence is limited, this report highlights the importance of preoperative assessment of corneal transparency and intraoperative visualization.

From a practical standpoint, eyes with visually significant corneal edema or opacity may be better managed with cataract surgery alone, staged glaucoma surgery, or another procedure that does not require the same degree of angle visualization. When MIGS is still considered, surgeons should prepare for the possibility that the glaucoma procedure may need to be abandoned intraoperatively if the angle cannot be safely identified.

### 9.5. Device-Related Considerations

Stent-based Schlemm canal procedures involve permanent device implantation. Therefore, device-related considerations include malposition, obstruction, under-implantation, over-implantation, iris contact, and limited access to functional collector channels [22,23,26,27,28,29,30,31,32]. Hydrus microstent additionally scaffolds a segment of Schlemm canal, and correct placement within Schlemm canal is essential [26,27,29,31,32]. Although clinically significant device-related complications are uncommon, they should be considered during surgical planning and postoperative gonioscopic evaluation.

Another practical issue is MRI compatibility. Some stent-based Schlemm canal devices, including iStent inject/iStent inject W and Hydrus Microstent, are MR Conditional under device-specific conditions according to manufacturer labeling and instructions for use [89,90]. According to device labeling, iStent/iStent inject/iStent inject W and Hydrus Microstent are MR Conditional under specified conditions, including a static magnetic field of 3 T or less, a maximum spatial field gradient of 4000 gauss/cm (40 T/m), and a whole-body averaged SAR of 4 W/kg, although exact conditions should be confirmed from the relevant device-specific labeling [89,90,91]. Patients should be informed that they have an implanted glaucoma device and should notify medical staff before undergoing MRI.

### 9.6. Balancing Safety and Efficacy

The overall safety profile of Schlemm canal-based MIGS remains favorable compared with conventional filtering surgery [16,18,19,20]. However, the safety advantage should not lead to oversimplification. Stent-based Schlemm canal procedures generally have a lower hyphema risk, but their IOP-lowering effect may be modest. Trabeculotomy/goniotomy-based procedures may provide comparable or greater IOP reduction in selected eyes, but hyphema, IOP spikes, and rare hypotony-related complications require closer consideration. Therefore, the choice of procedure should reflect the desired IOP reduction, medication burden, tolerance for early postoperative visual disturbance, angle visibility, bleeding risk, refractive goals, and the patient’s potential need for future filtration surgery [22,23,25,26,27,28,29,30,31,32,36,41,42,43,44,45,46,47,48,49,50,92,93,94].

## 10. Patient Selection and Surgical Planning

The decision to combine MIGS with cataract surgery should be individualized. A patient using glaucoma medications is not automatically an ideal candidate for combined MIGS, and conversely, some patients with well-controlled IOP may benefit substantially from medication reduction. The key question is not simply whether the patient has glaucoma, but what the surgeon aims to achieve after surgery.

### 10.1. Importance of Glaucoma Subtype and Angle Configuration

Glaucoma subtype and angle configuration should be considered before selecting combined cataract surgery and MIGS [1,55]. In eyes with open-angle glaucoma, normal-tension glaucoma, pseudoexfoliation glaucoma with an open angle, or steroid-induced glaucoma, Schlemm canal-based MIGS may be considered when the target IOP is compatible with a mid-teens range (approximately 13–17 mmHg) and medication reduction is clinically meaningful. In contrast, in eyes with narrow angles, primary angle closure, or primary angle-closure glaucoma, the effect of cataract surgery or lens extraction alone should first be considered, because lens extraction can deepen the anterior chamber and widen the angle [2,3,4,5,6,76,77]. When residual peripheral anterior synechiae, insufficient IOP control, or persistent medication burden remains a concern after lens-related mechanisms have been addressed, goniosynechialysis-assisted trabeculotomy or goniotomy may be considered in selected eyes with adequate visualization [97]. Recent systematic review and meta-analysis evidence also supports the potential role of phacoemulsification combined with trabecular meshwork- or Schlemm canal-based MIGS in selected eyes with primary angle-closure glaucoma, although this evidence should be interpreted in relation to angle anatomy, residual synechial closure, and device-specific indications [66]. Trabecular micro-bypass stent procedures require careful confirmation of device-specific indications and contraindications, because many such devices are indicated for open-angle glaucoma and are contraindicated or not established in angle-closure glaucoma [90,91].

### 10.2. Good Candidates for Combined Cataract Surgery and MIGS

The most suitable candidates are patients with mild-to-moderate open-angle glaucoma whose postoperative target IOP is approximately in the mid-teens (approximately 13–17 mmHg). In these eyes, Schlemm canal-based MIGS can provide additional IOP reduction beyond cataract surgery alone while preserving a favorable safety profile. Patients with multiple topical medications, ocular surface disease, poor adherence, or difficulty with instillation may particularly benefit from medication reduction [7,8,9,10,11,12,13,14,72,73,74].

Medication reduction is especially important in elderly patients. Long-term topical therapy may cause ocular surface toxicity, and adherence is often suboptimal [7,8,9,10,11,12,13,14,72,73,74]. Reducing the number of medications may improve tolerability, simplify treatment, and reduce chronic ocular surface stress. In some patients, this benefit may be more meaningful than the absolute magnitude of IOP reduction.

Eyes with clear corneas and good angle visibility are also better candidates [62]. Because Schlemm canal-based MIGS requires intraoperative gonioscopy, preoperative assessment should include corneal clarity, anterior chamber depth, angle anatomy, and the presence of peripheral anterior synechiae. When the cornea is clear and the angle is visible, both trabecular micro-bypass stent procedures and trabeculotomy/goniotomy-based procedures can be performed more safely and reproducibly.

### 10.3. Patients in Whom Cataract Surgery Alone May Be Sufficient

Cataract surgery alone may be reasonable in patients with stable glaucoma controlled with a single medication, minimal or no visual field progression, no major medication-related ocular surface symptoms, and no strong desire to reduce treatment burden [2,4,5,6,55,63,64,65]. Cataract extraction may produce modest IOP reduction, and in some eyes this may be adequate [2,4,63,64,65].

This approach may also be appropriate when angle visibility is poor or when the additional risks of MIGS outweigh the expected benefits. For example, if corneal opacity or edema prevents reliable visualization of the trabecular meshwork, it may be safer to complete cataract surgery alone and reassess glaucoma management postoperatively.

### 10.4. Patients Who May Require Filtration Surgery

Schlemm canal-based MIGS is usually insufficient for patients with advanced glaucoma requiring low-teens or lower IOP (approximately 12 mmHg or lower). Because the conventional outflow pathway remains limited by distal outflow resistance and episcleral venous pressure, most Schlemm canal-based procedures tend to achieve postoperative IOP in the mid-teens rather than very low levels [26,27,61]. For eyes with severe visual field loss, fixation-threatening defects, or rapid progression despite apparently controlled IOP, a lower individualized target pressure may be required [1,55]. This concept is supported by AGIS, which demonstrated an association between lower postoperative IOP and reduced visual field progression [56]. In selected secondary glaucomas, such as uveitic glaucoma, trabeculectomy with mitomycin C remains an important option when substantial IOP reduction is required, although inflammation- and hypotony-related complications must be carefully considered [98]. Accordingly, filtration surgery should be considered when Schlemm canal-based MIGS is unlikely to achieve the required target IOP.

The decision between combined MIGS and filtration surgery is particularly important in patients undergoing cataract surgery. Combining cataract extraction with trabeculectomy may provide stronger IOP lowering but carries higher risks of hypotony, bleb-related complications, induced astigmatism, refractive shift, and more intensive postoperative management [57,58,59]. In contrast, combined cataract surgery and MIGS offers a safer and more refractively stable option but may not achieve a sufficiently low target IOP. Thus, the choice should be based on disease severity and target pressure rather than surgical convenience.

### 10.5. Choosing Between Stent-Based Schlemm Canal and Trabeculotomy/Goniotomy-Based Procedures

When MIGS is considered appropriate, the next question is which procedure should be selected. Stent-based Schlemm canal procedures may be preferred when low invasiveness, rapid recovery, lower risk of hyphema, and medication reduction are prioritized. These procedures are suitable for patients with mild-to-moderate glaucoma in whom modest additional IOP reduction is acceptable [22,23,26,27,28,29,30,31,32].

Trabeculotomy/goniotomy-based procedures may be considered when a greater IOP lowering effect is sought, although the expected benefit should be balanced against a higher probability of transient hyphema [25,35,36,41,42,43,44,45,46,47,48,49,50,51,52]. They may also be useful when permanent device implantation is not desired. However, surgeons should be prepared for early postoperative hyphema and IOP fluctuation. In eyes with high bleeding risk, anticoagulation, monocular status, advanced field loss, or occupations requiring rapid visual recovery, the risk-benefit balance should be discussed carefully.

### 10.6. Refractive Planning in Selected Eyes

Refractive planning should be integrated into patient selection. Most Schlemm canal-based MIGS procedures do not substantially worsen refractive outcomes [37,38,54], but eyes with high preoperative IOP or anticipated large IOP reduction, particularly after trabeculotomy-based surgery, may experience mild axial length shortening and mild hyperopic shift [58,75]. However, when substantial IOP reduction is clinically required, glaucoma control generally takes priority over refractive precision.

In eyes with pseudoexfoliation, angle closure, zonular weakness, or unstable biometry, surgeons should be cautious when promising highly precise refractive outcomes [76,77,78]. Preoperative ocular surface optimization is also important because chronic topical therapy may cause ocular-surface disturbance that can compromise keratometry, IOL calculation, and postoperative visual quality [7,8,10,11,14,72].

### 10.7. Practical Decision Algorithm

A practical algorithm can be summarized as follows [1,55,56]. First, confirm glaucoma subtype, angle status, baseline IOP, individualized target IOP, expected IOP reduction from cataract surgery alone, medication burden, ocular surface status, disease stage, progression rate, angle visibility, and endothelial reserve. The key early decision is not disease stage alone, but whether the individualized target IOP is compatible with Schlemm canal-based surgery.

If the eye requires low-teens or single-digit IOP, particularly in the presence of fixation-threatening progression or high risk of central visual loss, Schlemm canal-based MIGS alone may be insufficient and filtration surgery, tube shunt surgery, or a glaucoma-specialist procedure should be considered. Conversely, if a postoperative IOP in the mid-teens is acceptable, Schlemm canal-based MIGS may be considered when medication reduction or additional IOP lowering is clinically meaningful and the angle can be safely visualized.

Procedure selection should distinguish stent-based Schlemm canal MIGS from trabeculotomy/goniotomy-based MIGS. Stent-based Schlemm canal MIGS is best suited for eyes with mild-to-moderate open-angle glaucoma undergoing cataract surgery when IOP is medically controlled or near the individualized target, the postoperative target IOP can remain in the mid-teens, and the angle can be safely visualized. The main goals are medication reduction, ocular surface improvement, refractive stability, early visual recovery, low invasiveness, and a lower hyphema risk.

By contrast, trabeculotomy/goniotomy-based MIGS should not be selected or excluded according to disease stage alone. These procedures may be considered across a broader range of disease stages when the individualized target IOP is compatible with mid-teens pressure, when baseline IOP or medication burden provides room for clinically meaningful reduction, and when the angle can be safely visualized. For example, an eye with more advanced visual field loss but IOP above 20 mmHg may still be a reasonable candidate for initial trabeculotomy/goniotomy-based surgery if the intended postoperative target is in the mid-teens and conjunctival preservation is desirable. However, if the target IOP is in the low teens or single digits, or if fixation-threatening progression requires very low IOP, filtration surgery, tube shunt surgery, or glaucoma-specialist surgery should be considered rather than relying on Schlemm canal-based MIGS alone.

Medication burden should be operationalized rather than interpreted as the simple presence of any topical therapy [7,8,9,10,11,12,13,14,72,73,74]. Combined cataract surgery and MIGS is more compelling when the patient uses two or more glaucoma medications or medication classes, or when drop-related problems are clinically important even with fewer medications, such as ocular surface disease or preservative-related toxicity, medication intolerance, poor adherence, difficulty with instillation, or a strong preference to reduce drops. A practical medication-reduction goal is reduction of at least one medication or medication class. Cataract surgery alone may be sufficient when the eye is stable on no or one medication, adherence is good, ocular surface disease is minimal, progression is absent or slow, and the expected IOP reduction from cataract surgery alone is likely to meet the individualized target IOP.

Anatomic suitability must also be assessed before adding angle-based MIGS. Clear corneal media, adequate gonioscopic visualization of the nasal trabecular meshwork over the intended treatment area, an open angle or limited/manageable PAS, sufficient endothelial reserve, and stable zonules favor combined surgery. Poor angle visibility, extensive PAS involving the intended treatment area, corneal edema or opacity, marked endothelial compromise, shallow anterior chamber, or unstable zonules should lower the threshold for cataract surgery alone, staged glaucoma surgery, or another procedure that does not require the same degree of intraoperative angle visualization.

## 11. Limitations

This review has several limitations. First, although the search strategy and selection process were expanded to improve transparency, this article remains a narrative review and not a systematic review or meta-analysis. Study selection was based on relevance to a predefined cataract surgeon-oriented clinical question and priority of evidence, but formal risk-of-bias assessment was not performed.

Second, much of the available evidence for Schlemm canal-based MIGS combined with cataract surgery is derived from retrospective studies, registry analyses, post-market surveillance studies, and heterogeneous real-world cohorts. Randomized evidence is strongest for selected trabecular micro-bypass stent and Hydrus microstent procedures, whereas evidence for trabeculotomy/goniotomy-based procedures, refractive outcomes, toric IOL use, and specific glaucoma subtypes is less standardized.

Third, MIGS procedures are heterogeneous. Stent-based procedures, KDB goniotomy, Tanito microhook ab interno trabeculotomy, ab interno trabeculotomy, and canaloplasty-based procedures differ in mechanism, tissue disruption, bleeding risk, and expected IOP reduction. Conclusions drawn from one procedure should not be generalized uncritically to another.

Fourth, patient populations differ among studies. Asian and Japanese cohorts include a higher proportion of NTG and low-baseline-IOP eyes than many Western trial populations. Therefore, the relative importance of medication reduction, modest IOP lowering, and avoidance of postoperative complications may differ across regions. 

Fifth, refractive outcomes are not uniformly reported, and standardized reporting of prediction error, SIA, axial length change, visual recovery, ocular surface status, and patient-reported outcomes is still lacking.

## 12. Future Perspectives

Despite the rapid expansion of MIGS, several important questions remain unanswered. First, most studies of combined cataract surgery and MIGS have focused primarily on IOP and medication reduction. Fewer studies have evaluated refractive outcomes, surgically induced astigmatism, visual function, contrast sensitivity, reading performance, or patient-reported outcomes. Because cataract surgery is fundamentally a visual rehabilitation procedure, future MIGS studies should include standardized visual, refractive, and patient-reported endpoints [38,79,80,82].

Second, prospective data are needed to clarify refractive prediction error after different MIGS procedures. Studies should stratify eyes by glaucoma subtype, baseline IOP, magnitude of IOP reduction, axial length, anterior chamber depth, zonular status, and IOL type. Such data would help identify patients at risk of postoperative hyperopic shift or refractive surprise.

Third, the effect of IOP reduction on axial length and refractive outcome deserves further investigation. Kanda and colleagues reported that IOP reduction after trabeculotomy may influence axial length and IOL selection [75]. Future studies could examine whether IOL calculation should account for anticipated IOP reduction, particularly in eyes with high baseline IOP or planned trabeculotomy-based surgery.

Fourth, Asian data are particularly important. Normal-tension glaucoma is common in Japanese and other Asian populations, and baseline IOP is often lower than in Western randomized trials [69,70]. Therefore, the clinical value of MIGS in these populations may lie more in medication reduction, ocular surface improvement, and treatment simplification than in large absolute IOP reduction. Future studies should evaluate outcomes using endpoints appropriate for low-baseline-IOP populations.

Fifth, anterior segment imaging may help refine surgical planning and postoperative assessment. Anterior segment optical coherence tomography, ultrasound biomicroscopy, and intraoperative imaging may improve the assessment of angle anatomy, corneal status, implant position, and procedure-related complications in MIGS [95]. In addition, anterior segment optical coherence tomography angiography may provide noninvasive information on episcleral vascular changes after trabecular bypass MIGS and on limbal, conjunctival, and intrascleral vascular remodeling after cataract incisions [99,100]. The integration of imaging into preoperative planning and postoperative assessment may improve procedure selection, wound-healing assessment, and postoperative management.

Finally, future research should focus on individualized decision-making. Rather than asking whether MIGS should be combined with cataract surgery in all glaucoma patients, studies should identify which patients benefit most from which procedure. Predictive models incorporating glaucoma severity, target IOP, baseline IOP, medication burden, ocular surface disease, corneal transparency, angle visibility, and refractive goals may help guide surgical planning and further operationalize guideline-based target IOP setting in cataract-MIGS decision-making [1].

## 13. Conclusions

The decision to combine Schlemm canal-based MIGS with cataract surgery should be based on explicit treatment goals rather than on the presence of topical therapy alone. Cataract surgery alone is often reasonable when glaucoma is stable, medication burden is low, and the expected IOP after phacoemulsification is likely to meet the individualized target. Combined cataract surgery and Schlemm canal-based MIGS is most appropriate when the individualized target IOP is compatible with Schlemm canal-based surgery, the angle can be safely visualized, and medication reduction or modest additional IOP lowering is clinically meaningful. When a low-teens or single-digit target IOP is required, especially in eyes with fixation-threatening progression, filtration surgery, tube shunt surgery, staged surgery, or a glaucoma-specialist procedure should be considered.

Procedure selection should distinguish stent-based procedures from trabeculotomy/goniotomy-based procedures. Stent-based MIGS may be favored in mild-to-moderate open-angle glaucoma when IOP is medically controlled or near target, the desired postoperative IOP is compatible with mid-teens pressure, the angle can be safely visualized, and medication reduction, low invasiveness, lower hyphema risk, refractive stability, and early visual recovery are priorities. Trabeculotomy/goniotomy-based MIGS should not be selected or excluded according to disease stage alone; it may be considered across a broader range of disease stages when the target IOP remains compatible with mid-teens pressure, baseline IOP or medication burden provides room for clinically meaningful reduction, and the angle can be safely visualized. Advanced visual field loss alone does not automatically mandate filtration surgery.

## Figures and Tables

**Table 1:** Classification and comparison of Schlemm canal-based MIGS and conventional filtration surgery.

Category	Representative Procedures	Main Mechanism	Relative IOP/Medication Effect	Early Safety Profile	Refractive Considerations
Stent-based Schlemm canal procedures	iStent, iStent inject, Hydrus microstent	Bypass the trabecular meshwork and/or scaffold Schlemm canal to enhance conventional aqueous outflow	Mild to moderate IOP reduction; often meaningful medication reduction [22,23,24,25,26,27,28,29,30,31,32,33,34]	Lower hyphema risk than trabeculotomy/goniotomy-based procedures; device malposition or obstruction may occur [22,23,24,25,26,27,28,29,30,31,32,35,36]	Favorable refractive stability in available cohorts [37,38]; toric IOL implantation may be feasible [39,40]
Trabeculotomy/goniotomy procedures	KDB goniotomy, Tanito microhook ab interno trabeculotomy	Incise or excise the trabecular meshwork and reduce trabecular outflow resistance	Comparable to or greater than stent-based Schlemm canal procedures in selected eyes; often meaningful medication reduction [25,35,36,41,42,43,44,45,46,47,48,49,50,51,52]	Hyphema is more frequent; transient IOP spikes should be considered [25,36,41,42,43,44,45,46,47,48,49,50]	Usually limited SIA [37,38,53,54], but early hyphema may delay visual recovery [25,36,41,42,43,44,45,46,47,48,49,50]
Conventional filtration surgery	Trabeculectomy	Create subconjunctival filtration pathway	Strong IOP reduction; may achieve low target IOP [55,56]	Higher risk of hypotony, bleb-related complications, and more intensive postoperative management [55,57,58,59]	Greater potential for induced astigmatism, biometric change, and refractive shift [57,58,59]; more suitable when very low target IOP is required [55,56]

**Table 2:** Refractive and visual outcomes relevant to Schlemm canal-based MIGS combined with cataract surgery.

Domain	Evidence Base	Evidence Context	Main Message	Cataract-Surgeon Caution
Refractive predictability	Mostly retrospective and selected MIGS cohorts [37,38,54], with contextual cataract-surgery refractive-risk data [76,77,78]	Stent- and trabeculotomy/goniotomy-based MIGS	MIGS-related refractive impact appears limited in available cohorts	Baseline ocular/anatomic risk factors may still reduce refractive predictability
SIA	Comparative observational data [37,53]	Schlemm canal-based MIGS and filtering surgery	Additional SIA appears limited compared with filtering surgery	Incision location and corneal status may modify risk
Toric IOLs	Small procedure-specific studies [39,40]	Selected phaco-MIGS cohorts	Toric IOL implantation may be reasonable	Evidence remains procedure-specific; reliable keratometry/biometry and careful patient selection are required
Axial length/hyperopic shift	Trabeculectomy/GDD and trabeculotomy data [58,75]	Greater relevance when IOP reduction is large	Substantial IOP decrease may be associated with axial length shortening and mild hyperopic shift	Consider possible mild hyperopic shift when substantial IOP reduction is anticipated
Visual recovery	Narrative, observational, and complication-related evidence [25,36,41,42,43,44,45,46,47,48,49,50,62,79,80,81,82,83]	Phaco-MIGS procedures	Most cataract-related visual recovery is preserved, but early delay may occur	Hyphema is the main early concern; IOP spikes may be poorly tolerated in fixation-threatening glaucoma
Ocular surface	Glaucoma medication, ocular-surface, and adherence literature [7,8,9,10,11,12,13,14,72,73,74]	Medication reduction and ocular-surface outcomes	Medication reduction may improve ocular surface status, visual quality, and satisfaction	Ocular-surface disturbance may compromise keratometry, IOL calculation, and postoperative visual quality
Overall interpretation	Heterogeneous, procedure-specific evidence	Stents and trabeculotomy/goniotomy differ	Additional refractive impact attributable to MIGS appears limited in selected eyes	Small residual refractive errors can matter when unaided visual expectations are high

Note: GDD = glaucoma drainage device; IOL = intraocular lens; IOP = intraocular pressure; MIGS = minimally invasive glaucoma surgery; phaco = phacoemulsification; SIA = surgically induced astigmatism.

**Table 3:** Complications and practical considerations relevant to cataract surgeons when combining Schlemm canal-based MIGS with cataract surgery.

Category	Examples/Mechanisms	Stent-Based Schlemm Canal Procedures	Trabeculotomy/Goniotomy-Based Procedures	Practical Implication
Early bleeding	Hyphema, blood reflux	May occur, but less frequent and usually mild [22,23,24,25,26,27,28,29,30,31,32,35,36]	More frequent [25,36,41,42,43,44,45,46,47,48,49,50]	Warn patients about transient blurred vision; avoid overpromising early visual recovery, particularly after trabeculotomy/goniotomy-based procedures
Postoperative IOP elevation	IOP spikes; retained OVD; hyphema/clot-related obstruction; inflammation or steroid response	May occur; reported rates vary by definition and study design [22,23,24,25,26,27,28,29,30,31,32,33,35,36,60]	May occur; hyphema is more frequent, and clot-related obstruction should be considered [25,36,41,42,43,44,45,46,47,48,49,50,52]	More concerning in advanced or fixation-threatening glaucoma [1,55,56]
Corneal/endothelial considerations	Corneal edema, low endothelial reserve, device proximity	Generally favorable endothelial profile, but device-specific long-term endothelial data should be considered [81,84,85,87,88]	No permanent implant; caution with poor visualization or difficult manipulation	Use caution in low-reserve eyes, balancing endothelial risk against glaucoma-control benefit
Angle visualization and procedural accuracy	Poor angle view, PAS, implantation difficulty, incomplete incision	May cause failed or suboptimal implantation	May cause incomplete incision, iris trauma, or cyclodialysis	Abandon angle-based MIGS if visualization is unsafe [62]
Device-related considerations	MRI status, device labeling, implant documentation	MR Conditional labeling should be checked [89,90,91]	No permanent implant for KDB/microhook	Give device information to patients and document implant type
Hypotony-related findings	Persistent hypotony, cyclodialysis, ciliochoroidal detachment	Not typical	Persistent hypotony associated with cyclodialysis/ciliochoroidal detachment has been reported [92,93,94]	Persistent hypotony requires AS-OCT or UBM evaluation and timely management [92,93,94,95]
Refractive considerations	SIA, refractive prediction error	Additional refractive impact appears limited in available cohorts [37,38]	Additional refractive impact appears limited in available cohorts [37,54]	Counsel that refractive impact is usually limited, but small residual refractive errors may still matter
Future surgery	Conjunctival preservation	No filtering bleb; conjunctiva preserved	No filtering bleb; conjunctiva preserved	Preserves options for future filtration surgery [16,17,18,19,20,21]

Note: Data are summarized qualitatively from representative clinical trials, observational studies, reviews, complication reports, guidelines, and device-specific labeling cited in the main text. AS-OCT = anterior segment optical coherence tomography; IOP = intraocular pressure; KDB = Kahook Dual Blade; OVD = ophthalmic viscosurgical device; PAS = peripheral anterior synechiae; SIA = surgically induced astigmatism; UBM = ultrasound biomicroscopy.

## Data Availability

Data sharing is not applicable to this article, as no new data were generated or analyzed in this review.

## Supplementary Materials

The following supporting information can be downloaded at: https://www.mdpi.com/article/10.3390/jcm15145503/s1, Figure S1: Literature identification and reference-selection framework; Table S1: Selected representative studies and evidence profile; Table S2: PubMed/MEDLINE search domains, contextual checks, and final-reference mapping.

## Abbreviations

Abbreviation	Full term
MIGS	minimally invasive glaucoma surgery
IOP	intraocular pressure
IOL	intraocular lens
KDB	Kahook Dual Blade
POAG	primary open-angle glaucoma
NTG	normal-tension glaucoma
OSD	ocular surface disease
SIA	surgically induced astigmatism
PAS	peripheral anterior synechiae
ECL	endothelial cell loss
ECD	endothelial cell density
EVP	episcleral venous pressure
