# Supplementary material for: Should Schlemm Canal-Based MIGS Be Combined with Cataract Surgery in Patients Receiving Topical Glaucoma Therapy? A Cataract Surgeon-Oriented Review
Source: J Clin Med. 2026 Jul 14;15(14):5503. doi: 10.3390/jcm15145503 (PMC13413391; doi:10.3390/jcm15145503)
Supplement: Supplementary file 1 [file jcm-15-05503-s001.zip › Supplementary_Figure_S1.pdf]

Supplementary Figure S1. Literature identification and reference-selection framework

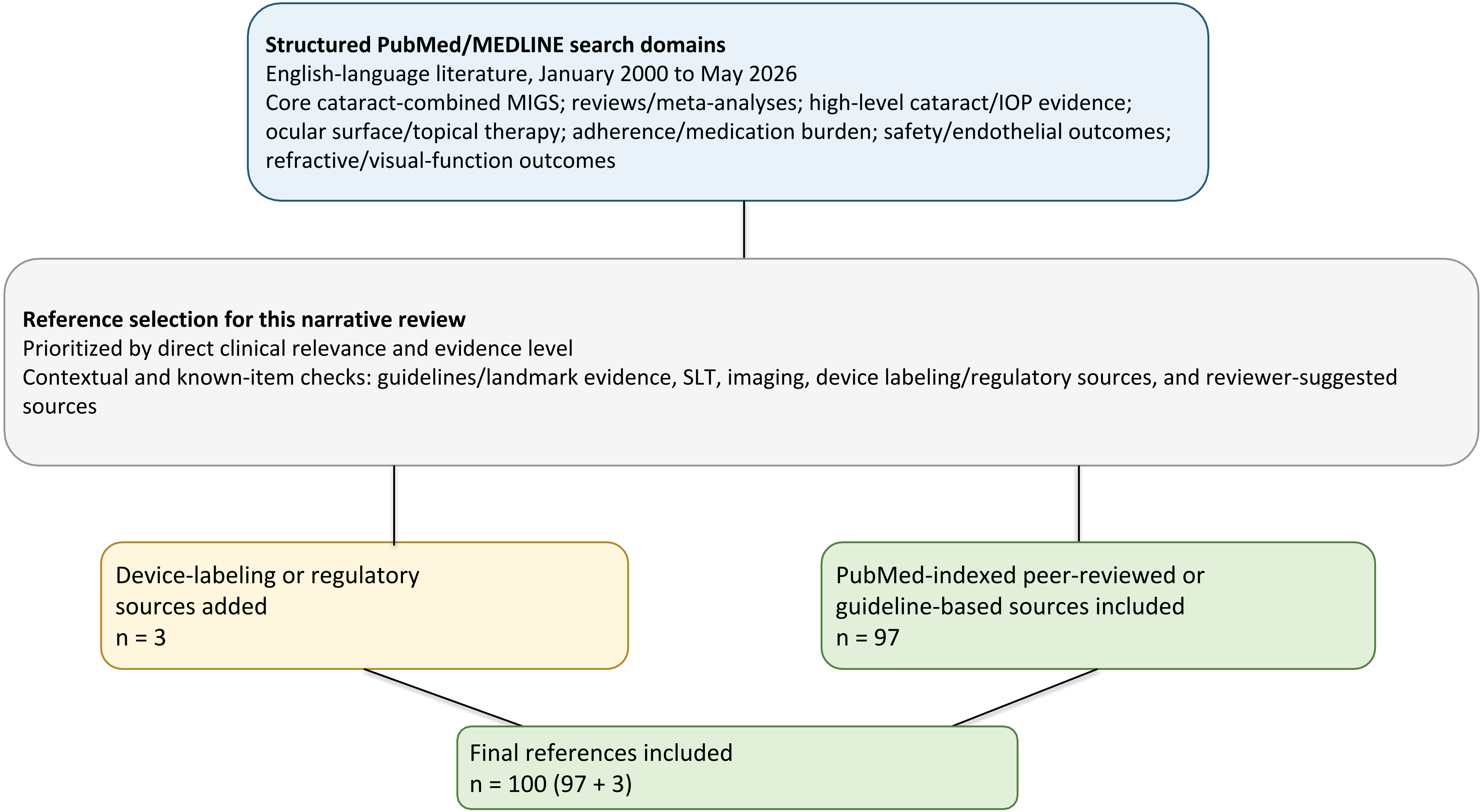

Note: This figure summarizes reference identification and selection for a narrative review. It is not a PRISMA flow diagram, and retrieved records were not used as systematic screening denominators.
