# Supplementary material for: Should Schlemm Canal-Based MIGS Be Combined with Cataract Surgery in Patients Receiving Topical Glaucoma Therapy? A Cataract Surgeon-Oriented Review
Source: J Clin Med. 2026 Jul 14;15(14):5503. doi: 10.3390/jcm15145503 (PMC13413391; doi:10.3390/jcm15145503)
Supplement: Supplementary file 1 [file jcm-15-05503-s001.zip › Table_S1.pdf]

Supplementary Table S1. Selected representative studies and evidence profile

Note: This table summarizes representative and high-priority sources used to support the cataract surgeon-oriented evidence synthesis. It is not intended to rank all cited studies or to serve as a formal risk-of-bias assessment. Evidence level and sample-size descriptors are qualitative because this article is a narrative review rather than a systematic review or meta-analysis.

Study / source	Procedure or topic	Study design / evidence level	Sample-size context	Population / baseline IOP context	Follow-up	Main finding	Major limitations	Cataract-surgeon implication
Japanese Glaucoma Society Guidelines [1]	Target IOP and treatment decision-making	Guideline	Guideline-based source	Glaucoma management in Japanese clinical practice	Not applicable	Glaucoma treatment should be guided by disease type, stage, and individualized target IOP.	Guideline statements are not procedure-specific evidence for cataract-combined MIGS.	Supports the central decision point that topical therapy alone is not an indication for adding MIGS; individualized target IOP is critical.
Shah et al.; Benekos et al.; Sabharwal et al. [63–65]	Cataract surgery alone and IOP reduction	Review / systematic review / meta-analysis	Evidence syntheses	Open-angle glaucoma, ocular hypertension, and cataract surgery populations	Variable	Cataract surgery alone can lower IOP, but the magnitude varies by baseline IOP and angle mechanism.	Heterogeneous populations and outcome definitions; not focused on direct MIGS selection.	Provides the comparator against which the incremental value of cataract-combined MIGS should be judged.
Samuelson et al. 2011 [22]	iStent + phacoemulsification	Randomized trial	Large RCT	Mild-to-moderate glaucoma with cataract	12 months	Greater IOP and medication reduction than cataract surgery alone.	Older first-generation device; selected trial population.	Established the incremental benefit of trabecular micro-bypass stent implantation combined with cataract surgery.
Samuelson et al. 2019 [23]	iStent inject + phacoemulsification	Prospective randomized pivotal trial	Large pivotal RCT	Mild-to-moderate POAG with cataract	24 months	Higher medication-free IOP reduction than cataract surgery alone.	Selected trial population; not designed to test very low target IOP achievement.	Supports second-generation trabecular micro-bypass stent use in selected cataract surgery candidates.
Gaskin et al. 2024 [34]	iStent inject + phacoemulsification vs phacoemulsification alone	Prospective randomized controlled trial	Moderate-sized independent RCT	Mild-to-moderate open-angle glaucoma with cataract	24 months	Greater medication reduction after combined surgery; IOP difference at 24 months was not significant.	No medication washout; selected OAG population.	Reinforces medication-burden reduction as a realistic goal of stent-based MIGS in medically treated cataract patients.
Samuelson et al. 2019; Ahmed et al. 2022 [26–27]	Hydrus microstent + phacoemulsification	Prospective randomized pivotal trial / long-term follow-up	Large multicenter RCT	POAG and cataract	2-5 years	Greater IOP/medication reduction and fewer subsequent glaucoma surgeries than cataract surgery alone.	Device-specific evidence; selected OAG population.	Provides strong long-term support for Hydrus combined with cataract surgery when the target IOP is compatible with Schlemm canal-based MIGS.
Montesano et al. 2023 [28]	Hydrus + phacoemulsification and visual-field outcomes	Post hoc / long-term analysis of RCT cohort	Large RCT-derived cohort	POAG and cataract	5 years	Long-term visual-field outcomes provided additional functional context for combined Hydrus surgery.	Analysis depends on trial population and available visual-field data.	Supports considering functional endpoints, not only IOP and medication count.
Tan et al. 2026 [68]	Hydrus versus iStent inject combined with cataract surgery	Long-term comparative cohort	Real-world comparative cohort	Open-angle glaucoma undergoing cataract-combined MIGS	Long-term follow-up	Reported comparative long-term outcomes between Hydrus and iStent inject combined with cataract surgery.	Nonrandomized; device selection may reflect surgeon and patient factors.	Useful when discussing device-specific expectations among stent-based MIGS procedures.
Barkander et al. 2023 [35]	iStent inject versus KDB with cataract surgery	Comparative observational study	Moderate retrospective cohort	Glaucoma patients undergoing cataract surgery	12 months	Both iStent inject and KDB combined with phacoemulsification reduced IOP; medication reduction was greater after KDB.	Nonrandomized; potential selection bias.	Illustrates the efficacy-safety trade-off between stent-based and goniotomy-based procedures.
Barkander et al. 2026 [60]	Cataract surgery alone versus iStent inject W or KDB Glide	Randomized controlled trial	Prospective RCT	Patients undergoing cataract surgery with glaucoma treatment indication	12 months	Directly compared cataract surgery alone with iStent Inject W or KDB Glide combined with cataract surgery; higher surgical success was mainly driven by medication reduction rather than greater IOP reduction.	Early-to-midterm follow-up; procedure-specific results require cautious generalization.	Highly relevant to the question of whether MIGS should be added at the time of cataract surgery.
Guedes et al. 2025 [36]	KDB goniotomy versus iStent combined with phacoemulsification	Systematic review / meta-analysis	Evidence synthesis	Open-angle glaucoma studies comparing or evaluating KDB and iStent with cataract surgery	Variable	Synthesized relative efficacy and safety of KDB and iStent approaches combined with cataract surgery.	Heterogeneity of included observational and trial data.	Supports disciplined evidence grading when comparing stent-based and goniotomy-based procedures.
Nakagawa et al. 2026 [25]	Trabecular micro-bypass stent versus ab interno trabeculotomy	Propensity score-matched retrospective study	Matched cohort	Japanese real-world open-angle glaucoma eyes	6 months	Similar short-term IOP outcomes; hyphema was more frequent after trabeculotomy.	Single-center; short follow-up.	Directly relevant to Japanese cataract-surgeon decision-making and safety counseling.
Dorairaj et al. 2018 [41]	KDB goniotomy + cataract surgery	Prospective / multicenter clinical study	Moderate clinical cohort	Medically treated glaucoma with cataract	12 months	Combined KDB and cataract surgery reduced IOP and medication burden.	No cataract-only randomized comparator; selected surgical cohort.	Provides core evidence for trabeculotomy/goniotomy-based MIGS combined with cataract surgery.
Maheshwari et al. 2026 [45]	Tanito microhook trabeculotomy + phacoemulsification	Prospective randomized trial	Prospective RCT	Open-angle glaucoma with cataract	2 years	Provided prospective randomized evidence for ab interno Tanito microhook trabeculotomy combined with cataract surgery.	Procedure- and population-specific; generalizability to other MIGS devices is limited.	Strengthens the evidence base for trabeculotomy-based MIGS beyond retrospective series.
Tanito et al. 2021 [43]	Microhook ab interno trabeculotomy	Large retrospective clinical series	Large initial clinical experience	Mixed glaucoma subtypes	Midterm follow-up	Reported broad clinical outcomes of microhook ab interno trabeculotomy.	Retrospective; not cataract-only comparative evidence.	Useful for understanding real-world effectiveness and limitations of microhook trabeculotomy.
Nakagawa et al. 2025 [51]	Two trabecular hooks for ab interno trabeculotomy	Propensity score-matched retrospective study	Matched cohort	Open-angle glaucoma undergoing trabeculotomy	Short-term follow-up	Compared two trabecular hook approaches with attention to clinical outcomes.	Single-center; retrospective design.	Supports procedure-selection discussion within trabeculotomy/goniotomy-based MIGS.

Study / source	Procedure or topic	Study design / evidence level	Sample-size context	Population / baseline IOP context	Follow-up	Main finding	Major limitations	Cataract-surgeon implication
Richter et al. 2024 [21]	Trabecular procedures combined with cataract surgery	American Academy of Ophthalmology report	Evidence report	Open-angle glaucoma and cataract surgery	Variable	Reviewed evidence for trabecular procedures combined with cataract surgery.	Evidence depends on available procedure-specific studies.	Provides authoritative support for the overall scope of cataract-combined trabecular MIGS.
Yuan et al. 2025 [17]	Combined MIGS with phacoemulsification in OAG	Systematic review / meta-analysis	Evidence synthesis	Open-angle glaucoma studies of combined MIGS and cataract surgery	Variable	Synthesized efficacy and safety of combined microinvasive glaucoma surgery with phacoemulsification.	Heterogeneous procedures and outcome definitions.	Provides review-level support for cataract-combined MIGS in OAG.
Fang et al. 2025 [66]	Phacoemulsification + TM/Schlemm canal-based MIGS in PACG	Systematic review / meta-analysis	Evidence synthesis	Primary angle-closure glaucoma with cataract	Variable	Suggested a potential role for adjunctive trabecular meshwork- or Schlemm canal-based procedures with phacoemulsification in selected PACG eyes.	PACG studies are heterogeneous and often differ from OAG MIGS studies.	Supports the angle-closure branch of the practical algorithm when PAS or residual outflow resistance persists.
Yu et al. 2025 [71]	MIGS in normal-tension glaucoma	Systematic review / meta-analysis	Evidence synthesis	Normal-tension glaucoma	Variable	Medication reduction or modest IOP lowering is often a more realistic goal than very low target IOP achievement.	Study heterogeneity and low baseline IOP limit generalization.	Supports cautious counseling in NTG and low-baseline-IOP eyes.
de Sousa Franco et al. 2026 [54]	GATT or KDB goniotomy combined with cataract surgery	Retrospective refractive-outcome study	Procedure-specific cohort	Cataract surgery combined with GATT or KDB goniotomy	Short- to midterm follow-up	Reported generally acceptable refractive predictability in selected eyes.	Retrospective; selected population; limited ability to isolate procedure effects.	Provides direct evidence for refractive counseling in KDB/GATT combined cataract surgery.
Nakagawa et al. 2025 [37]	SIA and refractive outcomes after cataract-combined MIGS	Retrospective comparative study	Real-world cataract-combined MIGS cohort	iStent, iStent inject W, and microhook trabeculotomy combined with cataract surgery	Postoperative refractive follow-up	SIA and refractive impact were generally limited across selected MIGS procedures.	Single-center; selected eyes; short- to midterm follow-up.	Supports cataract surgeons' refractive-planning perspective in MIGS candidates.
Shaheen et al. 2024 [38]	Refractive outcomes after combined cataract and MIGS	Retrospective cohort study	Large clinical cohort	Combined cataract and MIGS procedures	Postoperative refractive follow-up	Major refractive instability appeared uncommon in selected combined procedures.	Procedure mix and retrospective design limit device-specific conclusions.	Supports cautious reassurance regarding refractive predictability in selected eyes.
Kanda et al. 2024 [75]	IOP reduction, axial length, and IOL selection after trabeculotomy	Observational biometric study	Clinical cohort	Eyes undergoing trabeculotomy with IOL planning relevance	Short-term postoperative follow-up	IOP reduction may shorten axial length and influence IOL selection.	Biometric effect varies by magnitude of IOP reduction and baseline ocular features.	A secondary refractive consideration when large postoperative IOP reduction is expected, while recognizing that glaucoma control generally takes priority when substantial IOP reduction is clinically required.
Goto et al. 2024 [83]	Axial length and outcomes after cataract surgery with ab interno trabeculotomy	Retrospective clinical study	Clinical cohort	Cataract surgery combined with ab interno trabeculotomy	Short-term outcomes	Long axial length was associated with a smaller early postoperative IOP decrease and a higher incidence of early IOP spikes after phaco-LOT.	Short-term and procedure-specific; retrospective design.	Long-AL eyes may require closer early postoperative IOP monitoring after ab interno trabeculotomy.
Ichioka et al.; Lopez-Caballero et al. [39–40]	Toric IOL implantation with iStent + cataract surgery	Small procedure-specific clinical studies	Small to moderate cohorts	Glaucomatous eyes treated with toric IOLs and trabecular micro-bypass stents	Postoperative visual / refractive follow-up	Toric IOL implantation appeared feasible in selected glaucomatous eyes undergoing iStent-combined cataract surgery.	Small procedure-specific studies; findings should not be overgeneralized to complex eyes with PACG, XFG, zonular weakness, high IOP, or anticipated large IOP reduction.	Supports selective use of toric IOLs while avoiding overgeneralization to complex eyes.
Kasahara and Shoji 2025 [79]	Visual function after Schlemm canal-based MIGS	Narrative review	Review-level source	Schlemm canal-based MIGS studies	Variable	Visual function should be evaluated beyond IOP outcomes.	Review-level synthesis; procedure heterogeneity.	Supports including visual recovery and quality of vision in cataract-surgeon counseling.
Sarkisian et al. 2020 [80]	Visual outcomes after combined cataract surgery and MIGS	Review / update	Review-level source / expert committee perspective	Combined cataract surgery and MIGS procedures	Variable / not applicable	Reviewed visual outcomes after combined cataract surgery and MIGS and emphasized that visual recovery should be considered alongside IOP and medication outcomes.	Review-level synthesis; procedure heterogeneity and limited standardized visual-outcome reporting.	Supports patient counseling regarding postoperative visual recovery and visual expectations.
Au Eong et al. 2026 [82]	Glaucoma-specific quality of life after combined cataract and MIGS	Patient-reported outcome study	Clinical cohort	Patients undergoing combined cataract and MIGS procedures	Postoperative QoL follow-up	Combined procedures may improve glaucoma-specific quality of life, partly through reduced treatment burden.	Outcome interpretation may be influenced by cataract-related visual improvement and medication changes.	Supports medication-burden and patient-reported outcome considerations.
Fea et al. 2015 [81]	Endothelial cell loss after Hydrus + cataract surgery	Comparative clinical study	Clinical cohort	Combined Hydrus and cataract surgery versus phacoemulsification alone	6 months	At 6 months, endothelial changes after combined Hydrus cataract surgery were comparable with those after phacoemulsification alone in selected eyes.	Short-term follow-up; device-specific.	Supports endothelial-risk assessment in cataract-combined MIGS.
Obuchowska et al.; Seah et al.; Ahmed et al. [84–85, 87]	Corneal endothelial safety in MIGS	Reviews / safety analyses	Evidence syntheses and safety-focused sources	MIGS procedures with endothelial considerations	Variable	Endothelial effects vary by device, procedure, anatomy, and follow-up duration.	Heterogeneity of devices and study designs.	Supports preoperative endothelial reserve assessment, especially in corneal disease or shallow anterior chambers.
Lass et al. 2019 [88]	CyPass and endothelial cell loss	Long-term safety analysis	Large trial-related cohort	Phacoemulsification with or without CyPass microstent	5 years	Demonstrated clinically important endothelial cell loss associated with CyPass.	Suprachoroidal device; not directly generalizable to Schlemm canal-based MIGS.	Provides an important cautionary example for long-term device-related endothelial surveillance.

Study / source	Procedure or topic	Study design / evidence level	Sample-size context	Population / baseline IOP context	Follow-up	Main finding	Major limitations	Cataract-surgeon implication
Gillmann et al. 2026 [86]	Safety and complications in MIGS	Systematic and narrative review	Safety-focused evidence synthesis	MIGS procedures from 2014-2024	Variable	Summarized procedure-specific adverse events and complication patterns.	Heterogeneity across MIGS categories and reporting standards.	Supports balanced counseling regarding hyphema, IOP spikes, inflammation, corneal events, and rare complications.
Ishida et al.; Nakagawa and Ishii; Kudo et al.; Bothun et al. [92–94, 96]	Rare but clinically important trabeculotomy-related complications	Case reports / small series	Low-level but clinically informative evidence	Aphakic, microhook, and ab interno trabeculotomy-related settings	Variable	Reported events such as vitreous hemorrhage, persistent hypotony, serous choroidal/retinal detachment, and cyclodialysis.	Low evidence level; rare events; not incidence estimates.	Useful for surgical consent and for caution in high-risk eyes despite generally favorable MIGS safety profiles.
Kolko et al.; Kemer et al.; Gundersen et al. [10–12]	Ocular surface disease and glaucoma therapy / iStent surgery	Review / systematic review / prospective contralateral study	Mixed evidence sources	Patients using topical glaucoma therapy or undergoing iStent inject surgery	Variable	Topical glaucoma therapy can worsen ocular surface disease; reducing drops may improve treatment tolerance.	Not all ocular surface benefits are attributable to MIGS alone.	Supports medication reduction and ocular surface improvement as legitimate goals of combined cataract-MIGS.
Newman-Casey et al.; de Crom et al.; Cvenkel and Kolko; Quaranta et al. [13–14, 73–74]	Adherence, persistence, and treatment burden	Narrative review / overview / randomized adherence intervention / review	Mixed evidence sources	Patients receiving long-term glaucoma therapy	Variable	Adherence, persistence, technique, and treatment burden are major barriers in glaucoma management.	Not procedure-specific evidence for cataract-combined MIGS.	Supports considering MIGS when medication-burden reduction is clinically meaningful.

Abbreviations: IOP, intraocular pressure; MIGS, minimally invasive glaucoma surgery; OAG, open-angle glaucoma; POAG, primary open-angle glaucoma; PACG, primary angle-closure glaucoma; KDB, Kahook Dual Blade; GATT, gonioscopy-assisted transluminal trabeculotomy; SIA, surgically induced astigmatism; IOL, intraocular lens; QoL, quality of life; TM, trabecular meshwork; XFG, exfoliation glaucoma.
