# Supplementary material for: Should Schlemm Canal-Based MIGS Be Combined with Cataract Surgery in Patients Receiving Topical Glaucoma Therapy? A Cataract Surgeon-Oriented Review
Source: J Clin Med. 2026 Jul 14;15(14):5503. doi: 10.3390/jcm15145503 (PMC13413391; doi:10.3390/jcm15145503)
Supplement: Supplementary file 1 [file jcm-15-05503-s001.zip › Table_S2.pdf]

Supplementary Table S2. PubMed/MEDLINE search domains, contextual checks, and final-reference mapping

Database: PubMed/MEDLINE. **Search period:** January 1, 2000 to May 31, 2026. **Language:** English. **Animal-only exclusion filter:** NOT (animals[MeSH Terms] NOT humans[MeSH Terms]).

Note: This consolidated table lists the search domain or contextual check, role/purpose, approximate record count, and the final cited sources identified or prioritized through each domain. The reference-count column refers to the 97 PubMed-indexed peer-reviewed or guideline-based final sources unless otherwise specified; the 3 device-labeling or regulatory sources are indicated separately where applicable. Because search domains intentionally overlapped, the same final reference may appear in more than one row, and counts should not be summed. Contextual/known-item checks were not used as PRISMA-style screening denominators.

Domain	Role / purpose	Records	No. of final cited sources and reference numbers	Exact PubMed/MEDLINE string or contextual check
**S1**	Structured domain<br>Core cataract-combined Schlemm canal-based or trabecular meshwork-based MIGS	972	42: 17, 21–36, 41–52, 54, 60–62, 66–68, 71, 79–80, 82–83, 97	(("minimally invasive glaucoma surgery"[Title/Abstract] OR "microinvasive glaucoma surgery"[Title/Abstract] OR MIGS[Title/Abstract] OR iStent[Title/Abstract] OR "trabecular micro-bypass"[Title/Abstract] OR Hydrus[Title/Abstract] OR "Kahook Dual Blade"[Title/Abstract] OR KDB[Title/Abstract] OR "Tanito microhook"[Title/Abstract] OR microhook[Title/Abstract] OR trabeculotomy[Title/Abstract] OR goniotomy[Title/Abstract] OR "ab interno trabeculotomy"[Title/Abstract]) AND (cataract[Title/Abstract] OR phacoemulsification[Title/Abstract] OR "cataract surgery"[Title/Abstract] OR phaco[Title/Abstract]) AND glaucoma[Title/Abstract]) AND english[Language] AND ("2000/01/01"[Date - Publication] : "2026/05/31"[Date - Publication]) NOT (animals[MeSH Terms] NOT humans[MeSH Terms])
**S2**	Structured domain<br>Review-level evidence on Schlemm canal-based or trabecular MIGS	443	22: 11, 16–21, 31, 36, 61, 63, 65–67, 71, 73, 79–80, 84–86, 95	(("minimally invasive glaucoma surgery"[Title/Abstract] OR "microinvasive glaucoma surgery"[Title/Abstract] OR MIGS[Title/Abstract] OR "Schlemm canal"[Title/Abstract] OR "Schlemm's canal"[Title/Abstract] OR "trabecular micro-bypass"[Title/Abstract] OR trabeculotomy[Title/Abstract] OR goniotomy[Title/Abstract]) AND (review[Publication Type] OR "systematic review"[Title/Abstract] OR "meta-analysis"[Title/Abstract] OR "meta analysis"[Title/Abstract] OR review[Title]) AND glaucoma[Title/Abstract]) AND english[Language] AND ("2000/01/01"[Date - Publication] : "2026/05/31"[Date - Publication]) NOT (animals[MeSH Terms] NOT humans[MeSH Terms])
**S3**	Structured high-level evidence check<br>Cataract surgery, lens extraction, and IOP reduction, including high-level cataract-combined MIGS evidence	290	20: 2–6, 17, 21–23, 26–28, 33–34, 60, 63–67	(("cataract surgery"[Title/Abstract] OR phacoemulsification[Title/Abstract] OR "cataract extraction"[Title/Abstract] OR "lens extraction"[Title/Abstract]) AND ("intraocular pressure"[Title/Abstract] OR IOP[Title/Abstract]) AND (glaucoma[Title/Abstract] OR "ocular hypertension"[Title/Abstract] OR "angle-closure glaucoma"[Title/Abstract] OR "open-angle glaucoma"[Title/Abstract]) AND ("systematic review"[Title/Abstract] OR "meta-analysis"[Title/Abstract] OR "American Academy of Ophthalmology"[Title/Abstract] OR "randomized controlled trial"[Publication Type] OR trial[Title])) AND english[Language] AND ("2000/01/01"[Date - Publication] : "2026/05/31"[Date - Publication]) NOT (animals[MeSH Terms] NOT humans[MeSH Terms])
**S4A**	Structured contextual domain<br>Ocular surface disease, preservatives, and topical glaucoma therapy	419	6: 7–8, 10–12, 72	((glaucoma[Title/Abstract] OR antiglaucoma[Title/Abstract] OR "glaucoma medication"[Title/Abstract] OR "glaucoma medications"[Title/Abstract]) AND ("ocular surface disease"[Title] OR "ocular surface"[Title] OR "dry eye"[Title] OR preservative*[Title] OR "benzalkonium chloride"[Title] OR "limbal stem cell deficiency"[Title] OR "corneal epithelial"[Title]) AND ("glaucoma medication"[Title/Abstract] OR "glaucoma medications"[Title/Abstract] OR antiglaucoma[Title/Abstract] OR "topical therapy"[Title/Abstract] OR eyedrop*[Title/Abstract] OR prostaglandin*[Title/Abstract] OR latanoprost[Title/Abstract] OR bimatoprost[Title/Abstract] OR timolol[Title/Abstract])) AND english[Language] AND ("2000/01/01"[Date - Publication] : "2026/05/31"[Date - Publication]) NOT (animals[MeSH Terms] NOT humans[MeSH Terms])
**S4B**	Structured contextual domain<br>Medication burden, adherence, persistence, and treatment burden	617	5: 9, 13–14, 73–74	(glaucoma[Title/Abstract] AND (adherence[Title] OR persistence[Title] OR compliance[Title] OR "medication burden"[Title/Abstract] OR "treatment burden"[Title/Abstract]) AND (medication*[Title/Abstract] OR eyedrop*[Title/Abstract] OR "topical therapy"[Title/Abstract] OR treatment[Title/Abstract])) AND english[Language] AND ("2000/01/01"[Date - Publication] : "2026/05/31"[Date - Publication]) NOT (animals[MeSH Terms] NOT humans[MeSH Terms])
**S5**	Structured safety domain<br>Safety, complications, hyphema, IOP spikes, and corneal endothelial outcomes after Schlemm canal-based MIGS	1391	45: 16, 18–36, 41–52, 60, 66, 81, 84–88, 92–94, 96–97	(("minimally invasive glaucoma surgery"[Title/Abstract] OR "microinvasive glaucoma surgery"[Title/Abstract] OR MIGS[Title/Abstract] OR iStent[Title/Abstract] OR Hydrus[Title/Abstract] OR "Kahook Dual Blade"[Title/Abstract] OR KDB[Title/Abstract] OR "Tanito microhook"[Title/Abstract] OR microhook[Title/Abstract] OR trabeculotomy[Title/Abstract] OR goniotomy[Title/Abstract] OR "ab interno trabeculotomy"[Title/Abstract]) AND (safety[Title/Abstract] OR complication*[Title/Abstract] OR hyphema[Title/Abstract] OR "IOP spike"[Title/Abstract] OR "intraocular pressure spike"[Title/Abstract] OR hypotony[Title/Abstract] OR inflammation[Title/Abstract] OR "corneal edema"[Title/Abstract] OR "corneal endothelial"[Title/Abstract] OR "endothelial cell"[Title/Abstract] OR "endothelial cell loss"[Title/Abstract])) AND english[Language] AND ("2000/01/01"[Date - Publication] : "2026/05/31"[Date - Publication]) NOT (animals[MeSH Terms] NOT humans[MeSH Terms])
**S6**	Structured supporting domain<br>Refractive, visual-function, axial-length, astigmatic, toric-IOL, and patient-reported outcome literature relevant to cataract-combined MIGS or comparator glaucoma surgery	496	20: 30, 34, 37–40, 43, 53–54, 57–59, 61–62, 73, 75, 79–80, 82–83	((glaucoma[Title/Abstract]) AND ((("minimally invasive glaucoma surgery"[Title/Abstract] OR "microinvasive glaucoma surgery"[Title/Abstract] OR MIGS[Title/Abstract] OR iStent[Title/Abstract] OR Hydrus[Title/Abstract] OR "trabecular micro-bypass"[Title/Abstract] OR "Kahook Dual Blade"[Title/Abstract] OR KDB[Title/Abstract] OR "Tanito microhook"[Title/Abstract] OR microhook[Title/Abstract] OR trabeculotomy[Title/Abstract] OR goniotomy[Title/Abstract] OR "ab interno trabeculotomy"[Title/Abstract]) AND (cataract[Title/Abstract] OR phacoemulsification[Title/Abstract] OR "cataract surgery"[Title/Abstract] OR phaco[Title/Abstract] OR "intraocular lens"[Title/Abstract] OR IOL[Title/Abstract])) OR trabeculectomy[Title/Abstract] OR "tube shunt"[Title/Abstract] OR "glaucoma drainage device"[Title/Abstract] OR XEN[Title/Abstract]) AND ("refractive outcome"[Title/Abstract] OR "refractive outcomes"[Title/Abstract] OR "refractive error"[Title/Abstract] OR "prediction error"[Title/Abstract] OR "surgically induced astigmatism"[Title/Abstract] OR SIA[Title/Abstract] OR "axial length"[Title/Abstract] OR "hyperopic shift"[Title/Abstract] OR "toric intraocular lens"[Title/Abstract] OR "visual recovery"[Title/Abstract] OR "visual function"[Title/Abstract] OR "visual outcome"[Title/Abstract] OR "visual outcomes"[Title/Abstract] OR "patient-reported outcome"[Title/Abstract] OR "patient-reported outcomes"[Title/Abstract] OR "quality of life"[Title/Abstract])) AND english[Language] AND ("2000/01/01"[Date - Publication] : "2026/05/31"[Date - Publication]) NOT (animals[MeSH Terms] NOT humans[MeSH Terms])
**C2**	Contextual/known-item check<br>Guidelines, target IOP, staging, visual-field progression, epidemiology, and landmark evidence. Broad domain used mainly for guideline and landmark-evidence checks.	2854	5: 1, 55–56, 69–70	((glaucoma[Title/Abstract] AND (guideline*[Title/Abstract] OR "target intraocular pressure"[Title/Abstract] OR "target IOP"[Title/Abstract] OR "disease stage"[Title/Abstract] OR staging[Title/Abstract] OR "visual field progression"[Title/Abstract] OR epidemiology[Title/Abstract])) OR ("Japan Glaucoma Society"[Title/Abstract] OR "European Glaucoma Society"[Title/Abstract] OR "American Academy of Ophthalmology"[Title/Abstract] OR "Tajimi Study"[Title/Abstract] OR "Advanced Glaucoma Intervention Study"[Title/Abstract] OR AGIS[Title/Abstract])) AND english[Language] AND ("2000/01/01"[Date - Publication] : "2026/05/31"[Date - Publication]) NOT (animals[MeSH Terms] NOT humans[MeSH Terms])<br><br>This broad guideline/landmark-evidence search was used as a contextual/known-item check and was not treated as a formal screening denominator.
**C3**	Known-item/contextual checks<br>SLT, Hydrus versus iStent comparative evidence, AS-OCTA/imaging, uveitic glaucoma, refractive-risk references for angle-closure and pseudoexfoliation contexts, device-labeling or regulatory sources, and reviewer-suggested or other known-item sources.	Not counted as a formal denominator	8 among the 97 PubMed-indexed/guideline-based sources: 15, 76–78, 95, 98–100; plus 3 device-labeling or regulatory sources: 89–91 (11 final cited sources in total)	Representative examples: selective laser trabeculoplasty AND systematic review/meta-analysis; Hydrus AND iStent; anterior segment AND optical coherence tomography angiography; uveitic glaucoma AND trabeculectomy/filtration surgery; refractive error AND angle-closure glaucoma; pseudoexfoliation glaucoma AND refractive surprise; device-labeling, MRI safety, manufacturer, and regulatory sources. These checks were used when clinically relevant and were not counted as separate systematic screening denominators.

Abbreviations: AS-OCTA, anterior segment optical coherence tomography angiography; IOP, intraocular pressure; MIGS, minimally invasive glaucoma surgery; SLT, selective laser trabeculoplasty.
